# NAT10-mediated N4-acetylcytidine modification in KLF9 mRNA promotes adipogenesis

**DOI:** 10.1038/s41418-025-01483-x

**Published:** 2025-03-23

**Authors:** Xinxing Wan, Linghao Wang, Md Asaduzzaman Khan, Lin Peng, Xiaoying Sun, Xuan Yi, Zhouqi Wang, Ke Chen

**Affiliations:** 1 Department of Endocrinology, The Third Xiangya Hospital of Central South University, Changsha, Hunan PR China; 2 Department of Biochemistry and Microbiology, School of Health & Life Sciences, North South University, Dhaka, Bangladesh; 3 Department of Nephrology, The First Hospital of Changsha, Changsha, Hunan PR China

**Keywords:** Translational research, Gene regulation

## Abstract

Dysfunctional adipogenesis is a major contributor of obesity. N-acetyltransferase 10 (NAT10) plays a crucial role in regulating N4-acetylcysteine (ac4C) modification in tRNA, 18SrRNA, and mRNA. As the sole “writer” in the ac4C modification process, NAT10 enhances mRNA stability and translation efficiency. There are few reports on the relationship between NAT10 and adipogenesis, as well as obesity. Our study revealed a significant upregulation of NAT10 in adipose tissues of obese individuals and high-fat diet-fed mice. Furthermore, our findings revealed that the overexpression of NAT10 promotes adipogenesis, while its silencing inhibits adipogenesis in both human adipose tissue-derived stem cells (hADSCs) and 3T3-L1 cells. These results indicate the intimate relationship between NAT10 and obesity. After silencing mouse NAT10 (mNAT10), we identified 30 genes that exhibited both hypo-ac4C modification and downregulation in their expression, utilizing a combined approach of acRIP-sequencing (acRIP-seq) and RNA-sequencing (RNA-seq). Among these genes, we validated KLF9 as a target of NAT10 through acRIP-PCR. KLF9, a pivotal transcription factor that positively regulates adipogenesis. Our findings showed that NAT10 enhances the stability of KLF9 mRNA and further activates the CEBPA/B-PPARG pathway. Furthermore, a dual-luciferase reporter assay demonstrated that NAT10 can bind to three motifs of mouse KLF9 and one motif of human KLF9. In vivo studies revealed that adipose tissue-targeted mouse AAV-NAT10 (AAV-shRNA-mNAT10) inhibits adipose tissue expansion in mice. Additionally, Remodelin, a specific NAT10 inhibitor, significantly reduced body weight, adipocyte size, and adipose tissue expansion in high-fat diet-fed mice by inhibiting KLF9 mRNA ac4C modification. These findings provide novel insights and experimental evidence of the prevention and treatment of obesity, highlighting NAT10 and its downstream targets as potential therapeutic targets.

## Introduction

Obesity poses a significant threat to human health, often leading to life-threatening conditions such as diabetes, coronary heart disease, and hypertension [1, 2]. The underlying cause of obesity resides in impaired adipogenesis, a process that induces proliferation and hypertrophy of adipocytes. This pathological state amplifies lipogenesis, inviting the infiltration of inflammatory cells and augmenting oxidative stress, resulting in cellular dysfunction [3–5].

Post-transcriptional modification of mRNA is a crucial regulatory mechanism that governs various biological processes, including mRNA stability, precursor cleavage, polyadenylation, transport, and translation initiation [6–8]. Among the diverse modifications, N6-adenosine methylation (m6A), N1-adenosine methylation (m1A), and cytosine hydroxylation (m5C) are some of the most common [9–11]. N4-acetylcysteine (ac4C) modification of RNA was previously detected only in tRNAs and 18S rRNA [12, 13]. In 2018, a pivotal discovery by Arango et al. [14]. revealed the presence of ac4C modification in mRNA. Intriguingly, N-acetyltransferase 10 (NAT10) stands as the sole known “writer” responsible for mRNA ac4C modification. NAT10 modifies the mRNA of downstream target genes through ac4C, thereby influencing the stability of these mRNAs and their protein translation efficiency [14]. However, the role of NAT10 in adipogenesis and its contribution to obesity remains unclear, leaving an unexplored area of research.

The NAT10 protein is a highly conserved entity in both prokaryotes and eukaryotes, exhibits a unique mechanism of action [15]. Specifically, it acetylates the N4 nucleoside ac4C of the cytosine within the target gene’s mRNA, leading to the formation of an intramolecular hydrogen bond, this modification profoundly influences the spatial conformation of cytosine and its subsequent binding with guanosine, ultimately affecting the stability and translation efficiency of the target gene’s mRNA [16–18]. Notably, the acetylation sites mediated by ac4C in the target gene’s mRNAs are frequently located on the third base of the codon, often adhering to a characteristic “CXXCXXCXX” structure. This modification can occur across various regions of the mRNA, including the 5’-untranslated region (5’-UTR), coding sequence (CDS), and 3’-untranslated region (3’-UTR) [14, 19]. Since Arango et al.‘s groundbreaking discovery of mRNA ac4C modification in 2018 [14], numerous studies have emerged highlighting the significance of this modification, it has been implicated in diverse biological processes such as oocyte maturation, germ cell meiosis, tumor cell proliferation, bone differentiation, nerve injury, and even fatty acid metabolism in cancer cells [15, 20–25]. There is currently no report on whether Remodelin can prevent obesity by inhibiting NAT10 mediated mRNA ac4C modification. Remodelin, a specific inhibitor of NAT10 [26], has been found effective in treating malnutrition and ameliorating the cellular phenotype of Hutchinson Gilford premature aging syndrome (HGPS) [27]. Prior research has also revealed that Remodelin exerts its regulatory effects on target gene function by inhibiting the NAT10-mediated ac4C modification of downstream mRNA. This modulation has been observed to inhibit proliferation and promote osteogenic differentiation in multiple mouse experiments [25, 28–31]. However, to date, there is no report whether Remodelin can prevent obesity by targeting NAT10-mediated mRNA ac4C modification.

In our study, we observed a significant upregulation of NAT10 in adipose tissues of obese individuals and mice fed a high-fat diet, suggesting a pivotal role in promoting adipogenesis. Furthermore, through a combined approach of acRIP-sequencing (acRIP-seq) and RNA-sequencing (RNA-seq), we validated KLF9 as a downstream target gene of NAT10. Notably, downregulation of NAT10 led to a decrease in KLF9 mRNA stability, impeding the adipogenesis process via the CEBPA/B-PPARG signaling pathway.

## Results

### Upregulation of NAT10 in adipose tissues of obese patients and high-fat diet mice

**Fig. 1:**
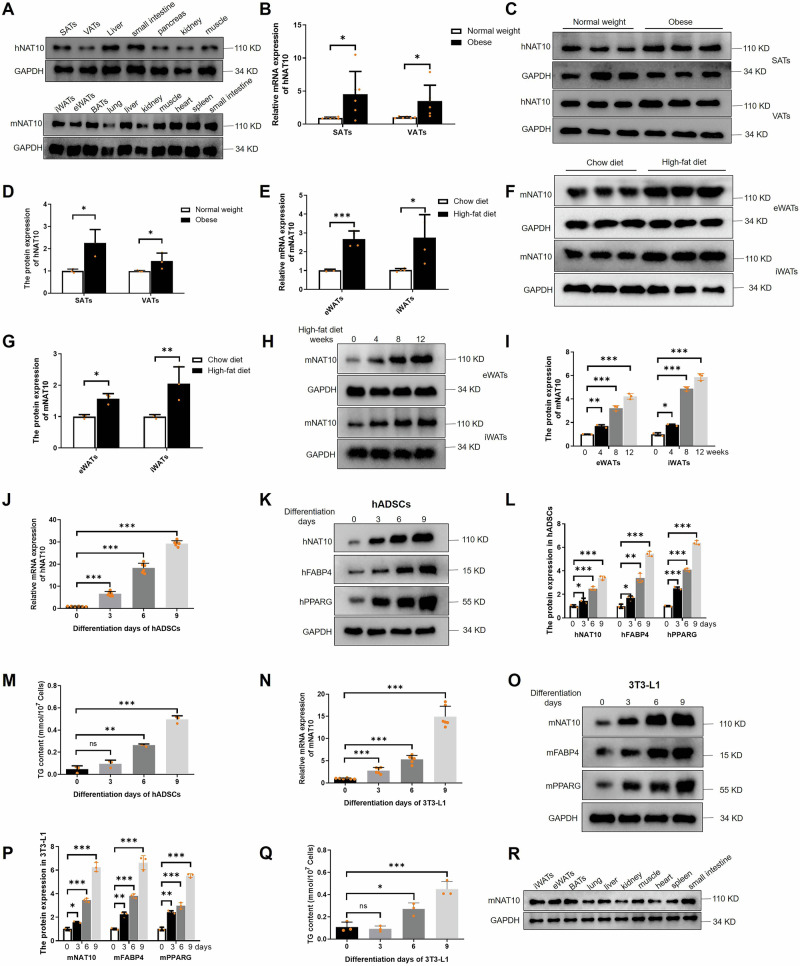
NAT10 is increased in adipogenesis. **A** Human NAT10 (hNAT10) and mouse NAT10 (mNAT10) were comprehensively distributed in different tissues of human and mice (*n* = 4 for each). **B** The hNAT10 mRNA (*n* = 6) and protein (*n* = 3). **C**, **D** was found increased in SATs and VATs of obese (*n* = 6) compared to normal weight patients (*n* = 6). **E** The mNAT10 mRNA (*n* = 4) and protein (n = 3) (**F**, **G**) was found elevated in eWATs and iWATs of high-fat diet (*n* = 4) than chew diet mice (*n* = 4). **H**, **I** The mNAT10 protein was gradually increased after mice were fed with high-fat diet for 12 weeks (*n* = 3). **J** hNAT10 mRNA expression was upregulated during hADSCs adipogenesis (*n* = 6). **K**, **L** The protein level expression of hNAT10, hFABP4 and hPPARG was upregulated during hADSCs adipogenesis (*n* = 3). **M** The TG content was found gradually increased during hADSCs adipogenesis (*n* = 3). **N** mNAT10 mRNA (*n* = 6) and protein expression of mNAT10, mFABP4 and mPPARG (n = 3) (**O**, **P**) were upregulated during 3T3-L1 adipogenesis (*n* = 3). **Q** The TG content was gradually increased during 3T3-L1 adipogenesis (*n* = 3). **R** The protein expression of mNAT10 were comprehensively distributed in mice differential tissues after fed with high-fat diet for 12 weeks (*n* = 4). *P < 0.05, **P < 0.01, ***P < 0.001.

To explore the hNAT10 expression, different tissues including subcutaneous adipose tissues (SATs), visceral adipose tissues (VATs), liver, small intestine, pancreas, kidney and muscle were collected from patients (*n* = 4), and our results showed the hNAT10 is comprehensively expressed. Moreover, for mNAT10 expression analysis, different tissues were isolated from mice (*n* = 4), also elucidated that mNAT10 is widely expressed in various tissues, including inguinal white adipose tissues (iWATs), epididymal white adipose tissues (eWATs), brown adipose tissues (BATs), the small intestine, and others (Fig. 1A). This implies that NAT10 has a broad tissue distribution. In addition, the hNAT10 mRNA (*n* = 6) and protein (*n* = 3) expression of SATs and VATs were found higher than in obese patients (BMI: >32 kg/m^2^) compared to normal individuals (BMI: 18.5–23.9 kg/m^2^) (Fig. 1B–D). Furthermore, in eWATs and iWATs of mice, the mNAT10 expression were also significantly upregulated in high-fat diet mice (*n* = 4) compared to chew diet (*n* = 4) (Fig. 1E–G). Nevertheless, the mNAT10 was gradually upregulated with high-fat diet for 0, 4, 8, and 12 weeks in eWATs and iWATs of mice (*n* = 3) (Fig. 1H, I). In addition, the hNAT10, mNAT10, the makers of adipogenesis including peroxisome proliferative activated receptor gamma (PPARG) and fatty acid binding protein 4 (FABP4) and cellular triglyceride (TG) content were gradually increased during hADSCs and 3T3-L1 adipogenesis (Fig. 1J–Q). Lastly, the mNAT10 expression in different tissues were detected after mice were fed with high-fat diet for 12 weeks (*n* = 4) (Fig. 1R).

### mRNA ac4C modification increased in adipogenesis

**Fig. 2:**
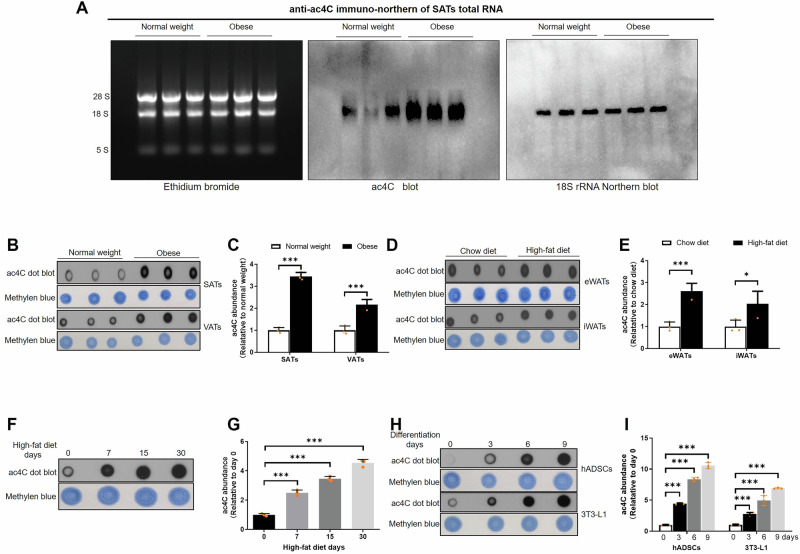
The level of mRNA ac4C modification is increased in adipogenesis. **A** Immune and northern blot revealed mRNA ac4C modification was found upregulated in SATs (*n* = 6). **B**, **C** mRNA ac4C modification was found upregulated in SATs and VATs of obese (*n* = 3) compared to normal weight patients (*n* = 3). **D**, **E** mRNA ac4C modification was found elevated in eWATs and iWATs of high-fat diet (*n* = 3) than chew diet mice (*n* = 3). **F**, **G** mRNA ac4C modification of iWATs was found increased after mice were fed with high-fat diet for 12 weeks (*n* = 3). **H**, **I** mRNA ac4C modification was upregulated during hADSCs and 3T3-L1 adipogenesis (*n* = 3). *P < 0.05, ***P < 0.001.

To explore mRNA ac4C modification in adipose tissues of obese patients, the mRNA ac4C modification in SATs and VATs of obese (*n* = 6) and normal weight (*n* = 6) patients were detected with anti-ac4C immuno-northern blot and ac4C dot blot. Our results showed that mRNA ac4C modification was drastically elevated in SATs and VATs of obese compared to normal weight patients (Fig. 2A–C). Besides, mRNA ac4C modification was also remarkably increased in eWATs and iWATs of mice fed with high-fat diet (*n* = 3) than chew diet (*n* = 3) for 12 weeks (Fig. 2D, E). Moreover, during high-fat diet, the mRNA ac4C modification was gradually enhanced in iWATs (*n* = 3) (Fig. 2F, G). In vitro, mRNA ac4C modification was gradually increased in hADSCs and 3T3-L1 cells adipogenesis (Fig. 2H, I). Our results implied that NAT10 mediated mRNA ac4C modification may play a critical role in adipogenesis.

### NAT10 promotes adipogenesis

**Fig. 3:**
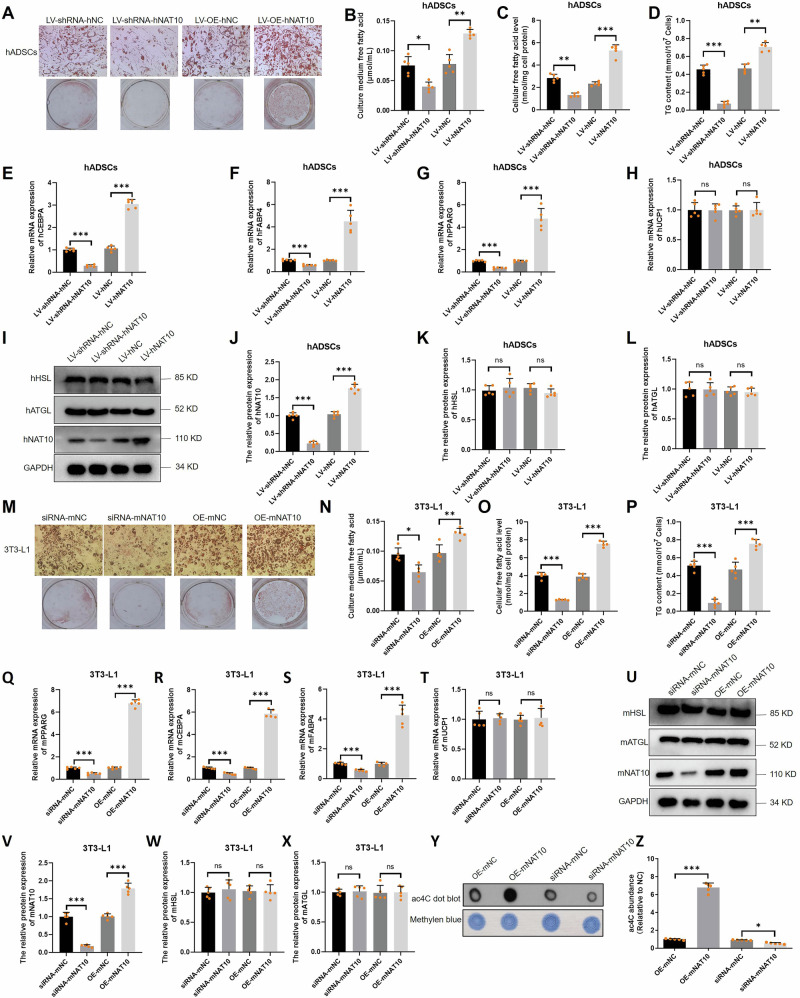
NAT10 promotes the mRNA ac4C modification and adipogenesis in vitro. **A** Overexpression and silencing of hNAT10 promoted or inhibited hADSCs adipogenesis (Oil red O staining, *n* = 5). Overexpression and silencing of hNAT10 promoted or inhibited FFA concentration in culture medium of hADSCs (*n* = 5) (**B**), cellular FFA contents (*n* = 5) (**C**), cellular TG contents of hADSCs (*n* = 5) (**D**). **E**–**G** Overexpression and silencing of hNAT10 promoted or inhibited the mRNA expression of hCEBPA, hFABP4 and hPPARG in hADSCs (*n* = 5). **H** Overexpression and silencing of hNAT10 could not promote or inhibit the mRNA expression of hUCP1 (*n* = 5) and protein expression of hHSL and hATGL in hADSCs (*n* = 5) (**I**–**L**). Overexpression and silencing of mNAT10 promoted or inhibited 3T3-L1 adipogenesis (Oil red O staining, *n* = 5) (**M**), FFA concentration in culture medium (**N**), cellular FFA contents (**O**) and cellular TG contents of 3T3-L1 (*n* = 5) (**P**). **Q**–**S** Overexpression and silencing of mNAT10 promoted or inhibited the mRNA expression of mPPARG, mCEBPA and mFABP4 in 3T3-L1 (*n* = 5). **T** Overexpression and silencing of mNAT10 could not promote or inhibit the mRNA expression of mUCP1 and protein expression of mHSL and mATGL in 3T3-L1 (*n* = 5) (**U–X**). **Y**, **Z** Overexpression and silencing of mNAT10 could promote or inhibit mRNA ac4C modification in 3T3-L1 (*n* = 5). *P < 0.05, **P < 0.01, ***P < 0.001.

To further investigate the role of NAT10 in adipogenesis, hADSCs and 3T3-L1 cells were overexpressed or silenced with NAT10, and the transfection efficiency of NAT10 were evaluated (Fig. S1). Next, after hADSCs were overexpressed or silenced with NAT10, the cells were induced to mature adipocytes. Oil Red O staining revealed that NAT10 markedly accelerated hADSCs adipogenesis. On the contrary, silencing NAT10 strongly prevented hADSCs adipogenesis (Fig. 3A). The FFA concentration in culture medium, cellular FFA and TG contents were significantly increased or decreased when hADSCs were overexpressed or silenced with NAT10 (Fig. 3B–D). In addition, CCAAT enhancer binding protein alpha (CEBPA), FABP4 and PPARG were upregulated or downregulated when hADSCs were overexpressed or silenced with NAT10 (Fig. 3E–G). However, the markers of BATs including uncoupling protein 1 (UCP1), hormone-sensitive lipase (HSL) and adipose triglyceride lipase (ATGL) were unchanged (Fig. 3H–L). In addition, increased and decreased NAT10 also significantly promoted or inhibited 3T3-L1 cells adipogenesis with Oil Red O staining and elevated or attenuated FFA concentration in culture medium, cellular FFA and TG contents (Fig. 3M–P), PPARG, CEBPA and FABP4 also were upregulated or downregulated when 3T3-L1 cells were overexpressed or silenced with NAT10 (Fig. 3Q–S), but the expression of UCP1, HSL and ATGL remained unchanged (Fig. 3T–X). Lastly, mRNA ac4C modification was also increased or decreased after 3T3-L1 cells were overexpressed or silenced with mNAT10 (Fig. 3Y, Z). These results indicated that NAT10 promotes human and mouse adipogenesis.

### NAT10 mediates mRNA ac4C modification in adipogenesis

**Fig. 4:**
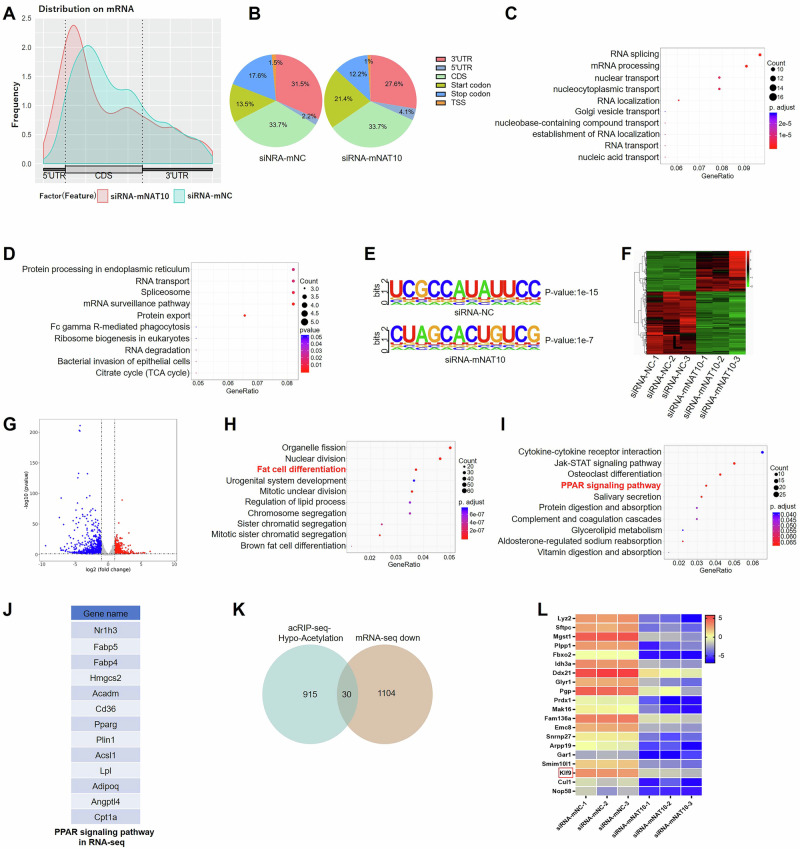
acRIP-seq and RNA-seq screening of the target genes of mNAT10. **A** acRIP-seq showed ac4C peaks distribution on mRNA. **B** acRIP-seq showed ac4C peaks proportion. **C** The GO-BP analysis of differential genes in siRNA-mNAT10 vs siRNA-mNC. **D** The KEGG pathway analysis of differential genes in siRNA-mNAT10 vs siRNA-mNC. **E** The highest abundance peaks of ac4C in two groups. **F** The hot map showed DEGs in RNA-seq. **G** The scatter plot showed DEGs in RNA-seq. **H** The GO analysis of DEGs in RNA-seq. **I** The KEGG pathway analysis of DEGs in RNA-seq. **J** 13 genes were enriched in PPAR pathway in RNA-seq. **K** The intersecting data in acRIP-seq and RNA-seq. **L** Hot map showed partial intersecting 20 genes in acRIP-seq and RNA-seq.

To find the target genes of NAT10 mediated mRNA ac4C modification in adipogenesis, after 3T3-L1 cells were transfected with siRNA-mNAT10 or siRNA-mNC for 48 h and then were induced differentiation for 48 h, the acRIP-seq combined with RNA-seq were used to screen the possible target genes, which both of mRNA were modified with ac4C, and expression were downregulated. In acRIP-seq, 1066 differential ac4C peaks were detected in siRNA-mNAT10 vs siRNA-mNC, the most of mRNA ac4C peaks were comprehensively distributed in 3’-UTR, CDS and 5’-UTR (Fig. 4A and supplementary table 3). In addition, in siRNA-mNAT10 and siRNA-mNC group, the highest abundance mRNA ac4C peaks were distributed in CDS (both were 33.7%) (Fig. 4B). The GO - biological process (GO-BP) analysis of the differential genes was enriched in RNA splicing, mRNA processing, nuclear transport etc (Fig. 4C). In KEGG pathway analysis, the differential genes were enriched in protein processing in endoplasmic reticulum, RNA transport, spliceosome etc (Fig. 4D). Moreover, most ac4C peaks contained typical “CXXCXXCXX” motifs and the highest abundance peaks of two groups are shown in Fig. 4E. In RNA-seq, the 1711 differential expression genes (DEGs) were detected in siRNA-mNAT10 vs siRNA-mNC, among them, 1104 DEGs were downregulated (Fig. 4F, G and supplementary table 4). The GO analysis showed that DEGs are enriched in fat cell differentiation, brown fat cell differentiation, and the regulation of lipid processes. etc (Fig. 4H). However, the KEGG pathway analysis of DEGs were enriched in cytokine-cytokine receptor interaction, JAK-STAT signaling pathway and PPAR signaling pathway etc (Fig. 4I). PPAR signaling pathway has been previously reported to be regulated by NAT10 through ac4C modification of genes such as ELOVL6, ACSL1, ACSL3, ACSL4, ACADSB, and ACAT1, thus modulating fatty acid metabolism in cancer cells [25]. Among them, 13 genes were participated in PPAR signaling pathway (Fig. 4J). There were 30 genes which were interacted of hypo-acetylation in acRIP-seq and downregulated DECs in RNA-seq (Fig. 4K), and partial results are showed in Fig. 4L. Our results indicated that NAT10-mediated mRNA ac4C modification strongly participated in adipogenesis.

### NAT10-mediates KLF9 mRNA ac4C modification regulates adipogenesis

**Fig. 5:**
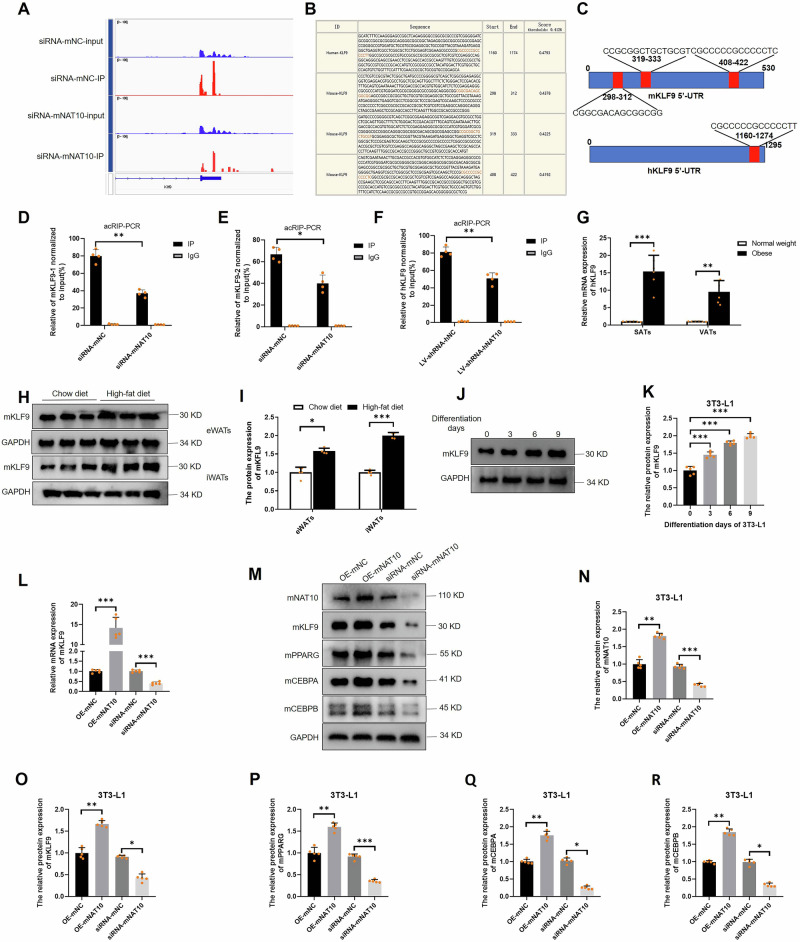
mNAT10 promotes adipogenesis and mKLF9 mRNA ac4C modification. **A** mKLF9 motif in acRIP-seq was visualized using IGV software. **B** The hNAT10 and mNAT10 ac4C motifs, predicted with PACES software. **C** hNAT10 and mNAT10 ac4C motifs in mRNA 5’-UTR. **D** acRIP-PCR verified that mNAT10 was able to ac4C modify mKLF9-1 (containing 2 motifs) (*n* = 4) and (**E**) mKLF9-2 (*n* = 4). **F** acRIP-PCR verified that hNAT10 was able to ac4C modify hKLF9 (*n* = 4). **G** The mRNA expression of hKLF9 was upregulated in SATs and VATs of obese (n = 6) compared to normal body weight (*n* = 6) patients. **H**, **I** The protein expression of mKLF9 was upregulated in iWATs and eWATs of high-fat diet group (*n* = 4) than chew diet group (*n* = 4). **J**, **K** mKLF9 was gradually upregulated during 3T3-L1 adipogenesis (*n* = 5). **L** mNAT10 positively regulated mKLF9 mRNA expression (*n* = 5). **M**–**R** mNAT10 positively regulated the protein expression of mKLF9, mPPARG, mCEBPA and mCEBPB in 3T3-L1 (*n* = 3). *P < 0.05, **P < 0.01, ***P < 0.001.

**Fig. 6:**
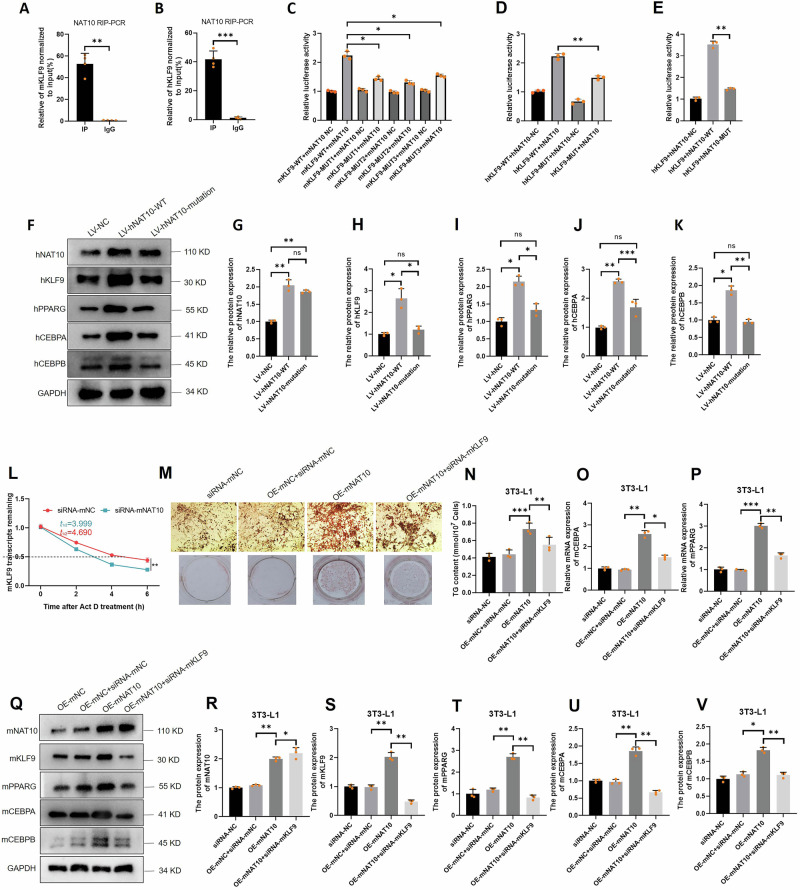
mNAT10 promotes adipogenesis via mKLF9 mRNA ac4C modification. **A** NAT10 RIP-PCR revealed that mNAT10 can interact with mKLF9 (*n* = 4). **B** NAT10 RIP-PCR revealed that hNAT10 can interact with hKLF9 (*n* = 4). Dual-luciferase reporter assay verified that mNAT10 is combined with 3 motifs of mKLF9 in 3T3-L1 cells (*n* = 3) (**C**)**;** hNAT10 combined with 1 motif of hNAT10 in HEK293T cells (*n* = 3) (**D**); hNAT10 ac4C modification site (G641E) mutation did not combine with hKLF9 in HEK293T cells (*n* = 3) (**E**). **F**–**K** hNAT10 ac4C modification site (G641E) mutation did not affect hKLF9, hPPARG, hCEBPA and hCEBPB (*n* = 3). **L** RNA decay experiment detected the mKLF9 mRNA stability after 3T3-L1 cells were silenced by mNAT10 for 48 h (*n* = 4). **M**, **N** The rescue experiments revealed that silencing of mKLF9 is able to partially decrease cellular lipid droplets form (Oil red O staining) (*n* = 3), and cellular TG content (*n* = 3). **O**, **P** The qPCR experiment revealed that the silencing of mKLF9 resulted in a reduction in the mRNA expression levels of both mCEBPA and mPPARG, subsequent to the overexpression of mNAT10 in 3T3-L1 cells (*n* = 3). **Q**–**V** The western blot experiment revealed that the silencing of mKLF9 resulted in a reduction in the protein expression levels of both mCEBPA, mCEBPB and mPPARG, subsequent to the overexpression of mNAT10 in 3T3-L1 cells (*n* = 3). *P < 0.05, **P < 0.01, ***P < 0.001.

To further explore the NAT10 mediated ac4C modification target genes in adipogenesis, we screened the interacted 30 genes of acRIP-seq and RNA-seq, and found mKLF9 might be a target gene which was modified by mNAT10 in Fig. 4L. KLF9 has been reported to be positively regulated in adipogenesis in 3T3-L1 cells via CEBPA/B-PPARG pathway [32, 33]. In acRIP-seq, mKLF9 motifs were visualized by IGV software (Fig. 5A). In addition, the hNAT10 and mNAT10 motifs also were predicted using PACES software, the results showed hKLF9 mRNA contained one motif might be ac4C modified too, however, there were three predicted motifs of mKLF9 (Fig. 5B). Both of hKLF9 and mKLF9 motifs are located at 5’-UTR (Fig. 5C). Next, we designed the specific primer according to motifs predicted by PACES software to detect whether the ac4C level of mKLF9 was regulated by the expression of mNAT10. The acRIP-PCR results showed that mKLF9 or hKLF9 mRNA were ac4C modified by mNAT10 or hNAT10, respectively (Fig. 5D–F). In addition, hKLF9 were significantly upregulated in SATs and VATs of obese (*n* = 6) compared to normal weight patients (*n* = 6) (Fig. 5G). Moreover, mKLF9 was also upregulated in iWATs of high-fat diet (n = 4) compared to chew diet mice (*n* = 4) (Fig. 5H, I). Nevertheless, mKLF9 was gradually increased during adipogenesis (Fig. 5J, K). To verify whether mNAT10 regulates adipogenesis via ac4C modification of mKLF9 mRNA and downstream mKLF9-mCEBPA/B-mPPARG pathway, firstly, the mKLF9 were detected after 3T3-L1 cells were overexpressed or silenced of mNAT10, our results revealed that mKLF9 mRNA and mKLF9, mCEBPA, mCEBPB and mPPARG protein expression were significantly upregulated or downregulated by overexpressed or silenced of mNAT10 (Fig. 5L–R). After conducting qPCR followed by NAT10 RIP (NAT10 RIP-PCR), we confirmed that both mKLF9 and hKLF9 mRNAs could interact with mNAT10 and hNAT10, respectively (Fig. 6A, B). Next, to verify which mKLF9 motif may participate in combining to mNAT10, the 3 mutated plasmids for 3 different motifs were constructed, and dual-luciferase reporter assay revealed that all of the 3 mutated motifs did not combine to mNAT10 (Fig. 6C). Our results confirmed that all 3 motifs of mKLF9 are ac4C modified. In addition, the hKLF9 mRNA was also verified to combine to hNAT10 using dual-luciferase report assay (Fig. 6D). Some previous studies have reported the no. 641 amino acid of hNAT10 is an ac4C acetylation site [30, 34, 35]. So, the mutated hNAT10 plasmid (G641E) was constructed to explore whether hNAT10 could regulate hKLF9 via hNAT10 acetylation site. Our results showed that the mutated hNAT10 did not combine with hKLF9 mRNA (Fig. 6E). In addition, mutated hNAT10 did not affect hKLF9-hCEBPA/B-hPPARG pathway (Fig. 6F–K). Next, to verify whether mNAT10 regulates mKLF9 stability, after 3T3-L1 cells were silenced with mKLF9 and then were treated with actinomycin D (5 μg/mL) to examine RNA decay. The results indicated that silenced mNAT10 dramatically decreased mKLF9 stability (Fig. 6L). To further verify whether mNAT10 regulated adipogenesis through mKLF9, the rescue experiment was performed, silencing of mKLF9 was able to partially decrease cellular lipid droplets form (Oil red O staining) (*n* = 3) (Fig. 6M), and partially decrease the cellular TG content (*n* = 3) (Fig. 6N), the qPCR experiment revealed that the silencing of mKLF9 resulted in a reduction in the mRNA expression levels of both mCEBPA and mPPARG, subsequent to the overexpression of mNAT10 in 3T3-L1 cells (*n* = 3) (Fig. 6O, P), and protein expression levels of both mCEBPA, mCEBPB and mPPARG also were decreased after overexpression of mNAT10 in 3T3-L1 cells (*n* = 3) (Fig. 6Q–V), these results verified the NAT10 regulated PPARG-CEBPA/B pathway in a KLF9-dependent manner. In summary, NAT10 promotes adipogenesis via KLF9 mRNA ac4C modification.

### Remodelin inhibits adipogenesis via ac4C KLF9 mRNA

**Fig. 7:**
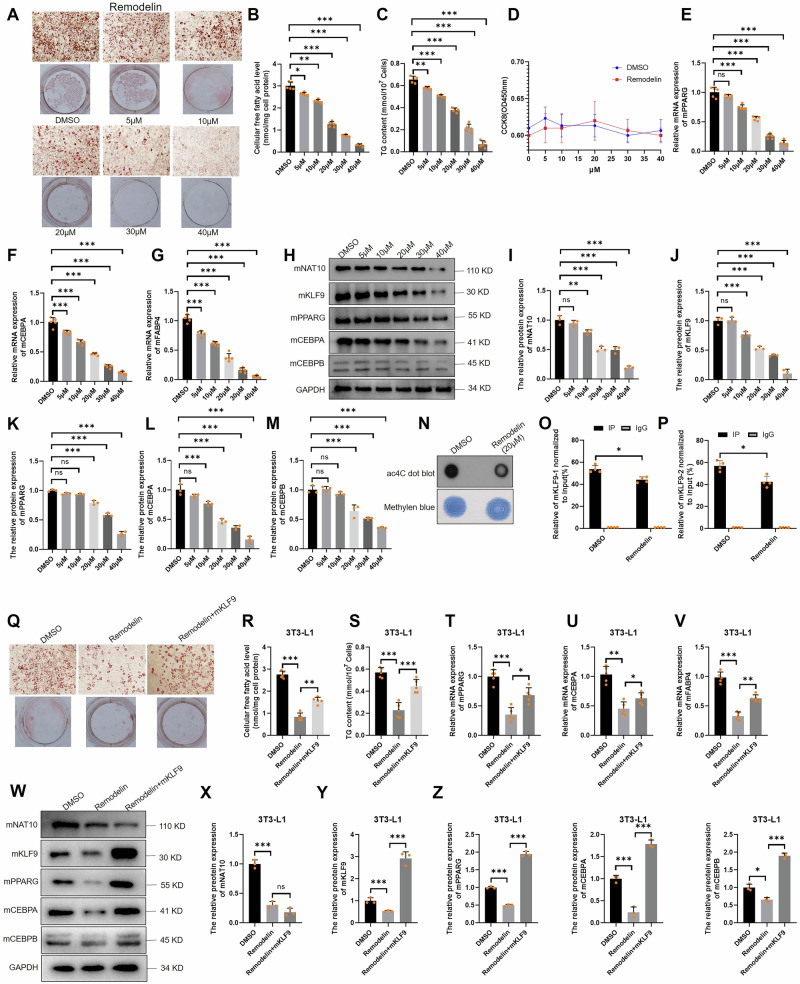
Remodelin inhibits adipogenesis through mNAT10-mKLF9-mCEBPA/B-mPPARG pathway. **A** Remodelin impeded adipogenesis (Oil red O staining) in a dose-depend manner (0–40 μM). **B** Remodelin impeded cellular FFA contents and TG contents (**C**) in a dose-depend manner (*n* = 5), but Remodelin did not affect cell viability (CCK-8) (*n* = 5) (**D**). **E**–**M** Remodelin significantly inhibited mRNA (*n* = 5) and protein (*n* = 3) expression of mPPARG, mCEPBA and mFABP4 (*n* = 5). **N** Ac4C dot blot showed that 20 μM Remodelin decreased 3T3-L1 cell’s mRNA ac4C modification. Remodelin inhibited mKLF9 (motif 1 and 2) (**O**) and (motif 3) mRNA ac4C modification (**P**), which was detected by using acRIP-PCR (*n* = 4). **Q**–**S** Overexpression of mKLF9 partially increased adipogenesis (Oil red O staining), cellular FFA contents and TC contents after 3T3-L1 cells were treated with 20 μM Remodelin (*n* = 5). **T**–**Z** Overexpression of mKLF9 partially increased the mRNA (*n* = 5) and protein (*n* = 3) of mPPARG, mCEBPA and mFABP4 after 3T3-L1 cells were treated with 20 μM Remodelin. *P < 0.05, **P < 0.01, ***P < 0.001.

To explore whether a specific NAT10 inhibitor, Remodelin could regulate adipogenesis, 3T3-L1 cells were induced for 9 days, where the culture medium contained 0–40 μM Remodelin (DMSO as control), the Oil red O staining showed that adipogenesis was inhibited and cellular FFA and TG contents were significantly decreased by Remodelin in dose-depend manner (Fig. 7A–C). Next, the cell viability was further evaluated with Cell Counting Kit-8 (CCK-8) assay, and we found that Remodelin did not have significant effect on cell viability (Fig. 7D). The mRNA and protein levels of mNAT10, mKLF9, mCEBPA, mCEBPB and mPPARG and mFABP4 mRNA were significantly inhibited by Remodelin in dose-depend manner (Fig. 7E–M). Moreover, 20 μM Remodelin decreased mRNA ac4C modification, which was confirmed by using ac4C dot bolt (Fig. 7N and Fig. S2). Besides, 20 μM Remodelin inhibited ac4C mKLF9 mRNA according acRIP-PCR (Fig. 7O, P). Additionally, overexpression of mKLF9 could partially rescue Remodelin-inhibited adipogenesis (Oil Red O staining) and cellular FFA and TG contents and mKLF9-mCEBPA/B-mPPARG pathway (Fig. 7Q–Z). Our results indicated that Remodelin drastically inhibits adipogenesis thorough mKLF9 mRNA ac4C modification.

### Silencing of mNAT10 inhibits adipose tissues depots in vivo via downregulation of mKLF9-mCEBPA/B-mPPARG pathway

**Fig. 8:**
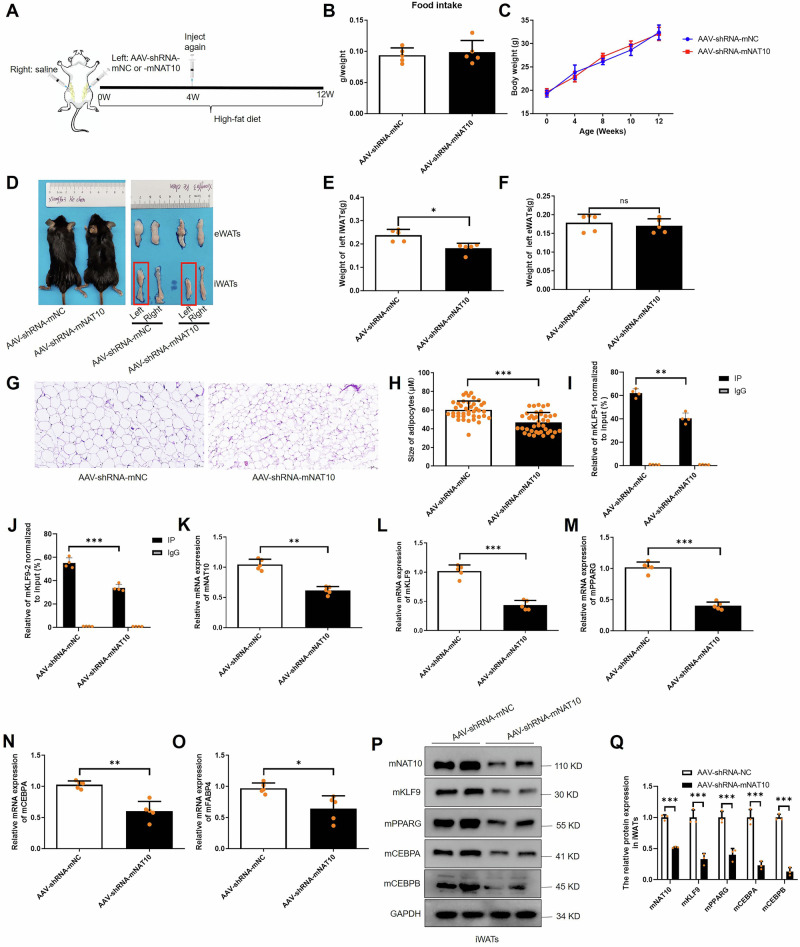
AAV-shRNA-mNAT10 inhibits adipose tissue expansion in vivo. **A** The protocol of AAV-shRNA-mNAT10 injection in mice. **B** Food intake in AAV-shRNA-mNAT10 and AAV-shRNA-mNC groups (*n* = 5). **C** Body weight in AAV-shRNA-mNAT10 and AAV-shRNA-mNC groups (*n* = 5). **D** The mice and eWATs and iWATs photos in AAV-shRNA-mNAT10 and AAV-shRNA-mNC groups. **E** The weight of iWATs and eWATs (**F**) decreased in the AAV-shRNA-mNAT10 group (*n* = 5). **G** H&E staining of iWATs in AAV-shRNA-mNAT10 and AAV-shRNA-mNC groups. **H** The adipocytes size of iWATs in AAV-shRNA-mNAT10 and AAV-shRNA-mNC groups (*n* = 40). **I** acRIP-PCR detected mKLF9 (motif 1 and 2) mRNA ac4C level in AAV-shRNA-mNAT10 and AAV-shRNA-mNC groups (*n* = 4). **J** acRIP-PCR detected motif 3 mRNA ac4C level in AAV-shRNA-mNAT10 and AAV-shRNA-mNC groups (*n* = 4). **K**–**O** The mNAT10, mKLF9, mPPARG, mCEBPA and mFABP4 mRNA expression in iWATs in AAV-shRNA-mNAT10 and AAV-shRNA-mNC groups (*n* = 5). **P**, **Q** The protein expression of mNAT10-mKLF9-mCEBPA/B-mPPARG pathway in iWATs (*n* = 3). *P < 0.05, **P < 0.01, ***P < 0.001.

The AAV injection experiments protocol in vivo are summarized in Fig. 8A, in which mice were fed high-fat diet for 12 weeks, and injected with adipose tissue targeted AAV-shRNA-mNAT10 (3 × 10^11^v.g, *n* = 5) or AAV-shRNA-mNC (3 × 10^11^v.g, *n* = 5) twice (at the beginning and on 4th week) in left iWATs. In addition, physiological saline was injected into the right iWATs twice (at the beginning and on 4th week). To evaluated tissue specificity of adipose tissue targeted AAV-shRNA-mNAT10 and AAV-shRNA-mNC, our western blot results showed that no reduction in the expression of mNAT10 in the heart, muscle, and liver in AAV-shRNA-mNAT10 compare to AAV-shRNA-mNC group (*n* = 6) (Fig. S3). In addition, our results showed that the food intake and body weight did not make any significant difference in two groups (Fig. 8B, C), however, the size and weight of left iWATs was smaller and lighter in AAV-shRNA-mNAT10 group compared to AAV-shRNA-mNC group (Fig. 8D, E), but the eWATs did not have any difference (Fig. 8F). In AAV-shRNA-mNAT10 group, the H&E staining showed that the adipocyte size of left iWATs was smaller in AAV-shRNA-mNAT10 group compared to AAV-shRNA-mNC group (Fig. 8G, H). Besides, GTT and ITT results showed no difference between the two groups (Fig. S4). In addition, the mKLF9 ac4C was found decreased in AAV-shRNA-mNAT10 group compared to AAV-shRNA-mNC group (Fig. 8I, J). Next, the mNAT10 and mKLF9-mCEBPA/B-mPPARG pathway were verified for downregulation in AAV-shRNA-mNAT10 group compared to AAV-shRNA-mNC group (Fig. 8K–O). Lastly, the protein expression pattern of mNAT10 and mKLF9-mCEBPA/B-mPPARG pathway are showed in Fig. 8P, Q. The above results suggested that mNAT10 inhibits adipose tissue depots in vivo.

### Remodelin impedes adipose tissue depots through inhibiting mNAT10 and further decreases mKLF9 ac4C modification

**Fig. 9:**
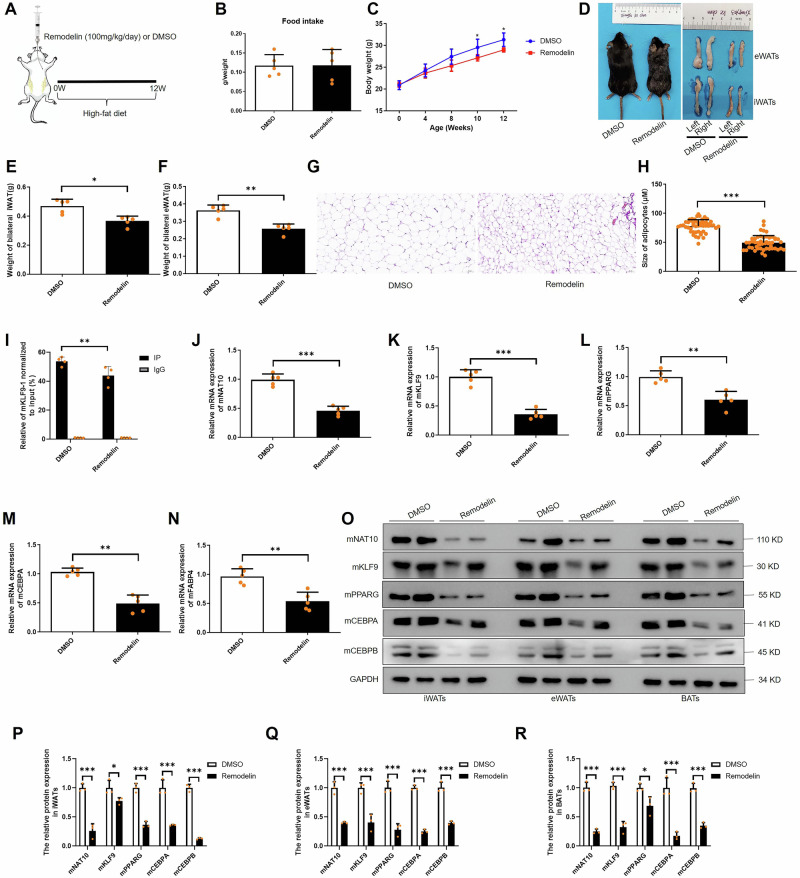
Remodelin decreases adipose tissue expansion in vivo through inhibiting mKLF9 ac4C modification. **A** The protocol of Remodelin gavage in mice. **B** Food intake in Remodelin and DMSO gavage groups (*n* = 5). **C** Body weight in Remodelin and DMSO gavage groups (*n* = 5). **D** The mice and eWATs and iWATs photos in Remodelin and DMSO gavage groups. **E** The weight of iWATs and eWATs (**F**) in Remodelin and DMSO gavage groups (*n* = 5). **G** H&E staining of iWATs in Remodelin and DMSO gavage groups. **H** The adipocyte size of iWATs in Remodelin and DMSO gavage groups (*n* = 50). **I** acRIP-PCR detected mKLF9 (motif 1 and 2) mRNA ac4C level in Remodelin and DMSO gavage groups (*n* = 4). **J**–**N** The mNAT10, mKLF9, mPPARG, CEBPA and FABP4 mRNA expression in Remodelin and DMSO gavage groups (*n* = 5). **O**–**R** The protein expression of mNAT10-mKLF9-mCEBPA/B-mPPARG pathway of iWATs, eWATs and BATs in Remodelin and DMSO gavage groups (*n* = 3). *P < 0.05, **P < 0.01, ***P < 0.001.

To further investigate whether Remodelin could inhibit adipogenesis via mNAT10 mediated downregulation of mKLF9, the mice were gavage with Remodelin (100 mg/kg/day) or DMSO and fed with high-fat diet for 12 weeks (Fig. 9A). Our results indicate that although the food intake did not show difference in Remodelin and DMSO groups (Fig. 9B), the body weight was significantly decreased in Remodelin group compared to DMSO group (Fig. 9C). In GTT and ITT experiments, our other study showed that Remodelin also elevated glucose tolerance and enhanced insulin sensitivity compared to DMSO group [36]. In addition, the size and weight of iWATs and eWATs were also apparently decreased in Remodelin group (Fig. 9D–F). The H&E staining showed that the adipocytes size of iWATs were smaller in Remodelin than DMSO group (Fig. 9G, H). In molecular level, Remodelin decreased mKLF9 mRNA ac4C modification (Fig. 9I) and dramatically inhibited mNAT10, mKLF9, mPPARG, mCEBPA and mFABP4 expression in iWATs (Fig. 9J–N). Lastly, Remodelin significantly inhibited the protein expression of mNAT10 and mKLF9-mCEBPA/B-mPPARG pathway in iWATs, eWATs and BATs (Fig. 9O–R). These results indicated that Remodelin prevents adipose tissue expansion via inhibiting mKLF9 mRNA ac4C modification.

## Discussion

While post-transcriptional modifications of mRNA, including m6A and m5C, have been documented to play a role in adipogenesis [11, 37, 38], the role of mRNA ac4C modification in regulating this process remains obscure and less explored. Our current research study reveals a novel finding: NAT10 enhances adipogenesis by facilitating ac4C modification of KLF9 mRNA, which subsequently leads to the upregulation of the CEBPA/B-PPARG pathway.

NAT10 is known to be widely expressed in various tissues of humans and mice, with particularly high levels in adipose tissues in mice [15, 39, 40]. While research on mRNA ac4C modification and its regulators is still limited, NAT10 stands out as the only known “writer” but not “eraser” and “reader” involved in this process, and its role in adipogenesis has not been extensively studied [41, 42]. Furthermore, the abundance of mRNA ac4C modification is relatively low compared to m6A methylation [14, 16]. Despite the scarcity of research, approximately 200 studies have investigated mRNA ac4C modification in different genes [43–46], yet the precise mechanisms underlying its regulation of mRNA function remain unclear. Consistent with most studies [47–49], our acRIP-seq analysis revealed that the majority of ac4C peaks are located in the CDS and 3’- UTR of mRNAs, with minimal distribution in the 5’-UTR (2.2% in the siNRA-mNC group and 4.1% in the siRNA-mNAT10 group). This aligns with previous findings that NAT10 primarily acetylates motifs located in the 3’-UTR, and that CDS acetylation positively regulates mRNA stability and translation efficiency [21, 50, 51]. Furthermore, Arango et al. observed that many motifs located in the 5’-UTR accumulate near the translation initiation site (TIS) [19]. However, in our study, the ac4C motifs of both human and mouse KLF9 genes were not found to be situated within the TIS region. It is generally believed that ac4C modification in the 5’-UTR can inhibit translation initiation, thereby suppressing protein translation and negatively regulating protein expression [19]. Nevertheless, in our research, we discovered that silencing NAT10 reduced KLF9 ac4C modification and impaired KLF9 mRNA stability. Intriguingly, Wu et al. also reported that NAT10 positively modulated transforming growth factor beta 1 (TGFB1) mRNA ac4C modification, mRNA stability, and protein expression, despite the TGFB1 mRNA ac4C motif being situated in the 5’-UTR as well [52]. Our further analysis revealed that the target for ac4C modification in KLF9 was actually the C base within the Kozak sequence, which occurred in the 5’-UTR outside the TIS region. This finding suggests that the location and context of ac4C modification, rather than its mere presence in the 5’-UTR, may determine its effect on mRNA and protein expression. Therefore, the question of how ac4C modification in the 5’-UTR specifically impacts mRNA and protein expression remains to be further investigated, particularly in cases where the modification does not occur near the TIS or Kozak sequence.

Our acRIP-PCR and dual-luciferase reporter assays revealed that NAT10 can bind to three motifs of mKLF9 and one motif of hKLF9. In a separate study, NAT10 was shown to modify distinct motifs within SYT9 mRNA [24]. These findings suggest that NAT10 has the ability to modify ac4C mRNA of target genes at multiple sites, rather than being restricted to a single site.

KLF9 is a member of KLFs family, which is a group of evolutionarily conserved and zinc finger structured transcription factors [53, 54]. KLF9 is reported to play a positive regulatory role in the adipogenesis of 3T3-L1 cells, at the molecular mechanism level, KLF9 can bind to the promoter of CEBPA, thereby promoting adipogenesis [32]. On the other hand, KLF9 also is able to interact with CEBPB and can form a complex to active PPARG2 transcript via combination of PPARG2 promoter [33]. Our study revealed that NAT10 was able to regulate adipogenesis through KLF9-CEBPA/B-PPARG pathway in vitro and in vivo.

Remodelin, a specific inhibitor of NAT10, has been extensively utilized to investigate the diverse biological functions of this enzyme [55]. In our study, Remodelin exhibited robust inhibitory effects on NAT10, significantly suppressing adipogenesis in a dose-dependent manner. In other research, Remodelin has been shown to alleviate cellular defects associated with HGPS [27], and to inhibit mitochondrial lipid metabolism in cancer cells [56]. While a previous study found that Remodelin did not affect mRNA ac4C modification [57], it is still widely used to target ac4C acetylation levels [23, 44, 58]. Both in vitro and in vivo studies have demonstrated that Remodelin can significantly hinder tumor progression [59–62]. Notably, several studies have shown that Remodelin is non-toxic to cells or mice [22, 55, 63, 64]. Consistent with these findings, in our study, concentrations of Remodelin ranging from 0 to 40 μM did not impact the viability of 3T3-L1 cells, and a 12-week gavage treatment with 100 mg/kg/day Remodelin did not lead to mortality in mice. However, it is worth mentioning that some mice exhibited partial whitening of their hair. Furthermore, there are indications that four FDA-approved drugs, including Fosaprepitant, Leucal, Fludarabine, and Dantrolene, may possess stronger inhibitory effects on NAT10 than Remodelin, in conclusion, further research is necessary to determine if Remodelin and these four drugs could potentially be used in the treatment of obesity through their ability to inhibit NAT10 [58].

In conclusion, we illustrated that human and mice NAT10 significantly promoted adipogenesis through KLF9 mRNA ac4C modification and increased KLF9 mRNA stability and further regulated KLF9-CEBPA/B-PPARG pathway. Furthermore, Remodelin might emerges as a promising small molecular inhibitor that holds potential for obesity treatment.

## Materials and methods

### Specimen collected

Human tissues were collected from different patients (*n* = 4) who suffered from abdomen trauma. The remaining tissues of SATs, VATs, liver, spleen and small intestine were obtained through surgery. The SATs and VATs were collected from individuals with normal body weight (*n* = 6, BMI: 18.5–23.9 kg/m^2^) and obese patients (*n* = 6, BMI: >32 kg/m^2^), the clinicopathological features of normal body weight and obese patients were provided in supplementary table 1. Different tissues including iWATs, eWATs, BATs, lung, liver, kidney, muscle, spleen and small intestine of C57BL/6 mice (*n* = 4) were collected too. In addition, C57BL/6 mice (*n* = 4) were fed by chow diet (Jiangsu Xietong Pharmaceutical Bio-engineering Co., Ltd., China, 1010001) and high-fat diet (XTHF60) (*n* = 4) for 12 weeks, eWATs, iWATs and BATs were isolated from mice at 0, 4, 8 and 12th week. This study was approved by the Ethics Committee of The Third Xiangya Hospital of Central South University, Changsha, China (NO: 2023-S027), and was performed according to the guidelines outlined in the Declaration of Helsinki. All participants provided written informed consent form. All animal experiments were performed according to the guidelines of the Ethical Committee for Animal Experiments of The Third Xiangya Hospital of Central South University (Changsha, China).

### hADSCs isolation and culture of 3T3-L1 cells

Approximately 10 g of SATs were obtained from patient’s VATs (*n* = 4), the adipose tissues were washed by PBS for three times, and cut into 1 × 1 mm^3^ size, then, the tissues were digested by collagenase I solution (Cat# 17100017, Gibco, Life Technology, Shanghai, China) for 1 h, the mixture were filtered with a 70-µm cell strainer (BD Falcon, Becton Dickinson, Franklin Lakes, NJ, USA), centrifuged at 150 × g for 10 min, gently poured out the supernatant, 3 ml of erythrocyte lysate (Beyotime Institute of Biotechnology, Shanghai, China) was used to remove red blood cells, centrifuged at 150 × g for 10 min again, the cells were washed with 10 ml PBS for three times and were cultured in DMEM/F12 (Cat#11320033, Gibco, Life Technology, Shanghai, China) containing 10% fetal bovine serum (Cat#A5670701, Gibco, Life Technology, Shanghai, China). Consistent with previous studies, we utilized cells from the fourth passage as hADSCs in our research [65–67]. 3T3-L1 cells were bought from Wellbiology (Changsha, China) and cultured with high glucose DMEM (Cat# 11965092, Gibco, Life Technology, Shanghai, China) containing 10% FBS and 100 U/ml penicillin and 100 μg/ml streptomycin.

### hADSCs and 3T3-L1 cells differentiation and Remodelin treatment

When hADSCs and 3T3-L1 cells grew in confluence, the cells were cultured in differentiation medium. For hADSCs, DMEM/F12 contained 1 μM dexamethasone (Cat# D4902), 10 μM insulin (Cat# I2643), 0.5 mM isobutylmethylxanthine (IBMX, Cat# I5879), and 200 μM indomethacin (Cat# I7378) all provided by Sigma, St. Louis, MO, USA). The medium was replaced every 2 days. For 3T3-L1 cells, DMEM contained 1 μM dexamethasone, 10 μM insulin and 0.5 mM IBMX, after 48 h, the medium was changed to DMEM containing 10 μM insulin for every 48 h. In addition, during hADSCs and 3T3-L1 cells differentiation, cells were treated with different concentration Remodelin (Cat#SD1168, Beyotime Institute of Biotechnology, Shanghai, China) (0, 5, 10, 20, 40 μM).

### Oil Red O staining and free fatty acid (FFA) measure

The differentiated adipocytes were fixed by 4% paraformaldehyde (Cat#P0099, Beyotime Institute of Biotechnology, Shanghai, China) for 4 h at room temperature and washed by PBS for 3 times, the Oil Red O staining (Cat#O0625, Sigma, St. Louis, MO, USA) was added to the plates and incubated at room temperature for 10 min and washed by water for 3 times. The images were captured by microscope (Leica DM IL LED Fluo, Germany). The culture medium and cellular FFA level were tested by fluorescent quantification kit (BC0590, Solarbio Science & Technology Co. LTD, Beijing, China).

### TG level measurement

TG was measured using the assay kit (BC0620, Solarbio Science & Technology Co., LTD, Beijing, China) following manufacturer’s guideline. In summary, 5 to 10 million cells were first collected into a centrifuge tube, and following centrifugation, the supernatant was discarded. Subsequently, 1 mL of reagent A was added to the cell precipitate, and the mixture was sonicated for 1 min at a power rate of 200 W, using a cycle of 2 s of ultrasound followed by 1 s of rest. After sonication, the mixture was centrifuged again at 8000 *g* and 4 °C for 10 min. Finally, the supernatant was collected and tested at a wavelength of 420 nm.

### Reverse transcription real-time quantitative PCR (RT-qPCR)

The RNA was isolated from cells and tissues using TRIzol (Cat#15596018CN, Invitrogen, Shanghai, China) method, and RNA was reverse transcribed to cDNA (Cat#K1622, Thermo Scientific, Shanghai, China), the qPCR amplification was performed with SYBR Green qPCR Mix (Cat#TSE201, Tsingke Biotech Co., Ltd., Beijing, China). The primer sequences are listed in supplementary table 2.

### Western blotting

The total cells or tissues protein were extracted using a protein extraction kit (Cat#KGB5303, KeyGEN BioTECH, Nanjing, China), protein concentration was quantified using BCA method (Cat#23225, Thermo Fisher, Shanghai, China), and 30 μg total protein samples were subjected to SDS-PAGE gel electrophoresis for 1.5 h. Separated proteins were then transferred from gel to nitrocellulose membrane at 300 mA for 1 h. Membrane was then blocked in 5% skimmed milk powder for 2 h. The primary antibody, such as NAT10 (13365-1-AP), PPARG (16643-1-AP), CEBPA (29388-1-AP), CEBPB (23431-1-AP), FABP4 (12802-1-AP), HSL (17333-1-AP), ATGL (55190-1-AP), and GAPDH (60004-1-Ig) [all provided by Proteintech, Wuhan, China] and KLF9 (sc-376422, Santa Cruz, Texas, USA) were used for the incubation of membrane for overnight. The secondary antibody (Cat#SA00001-1 and Cat#SA00001-2, Proteintech, Wuhan, China) was incubated for 1 h, and after washing, ECL reaction (Cat#32209, Thermo Fisher, Shanghai, China) was performed to develop image, which was observed by GelDoc XR gel imaging analysis system, and band was analyzed.

### Immuno-northern blot of RNA in SATs

The RNA extracted from SATs was purified through ethanol precipitation at a temperature of −80 °C for overnight. Subsequently, the precipitated RNA pellets were resuspended in a solution containing LET buffer (composed of 25 mM Tris-HCl at pH 8.0, 100 mM LiCl, and 20 mM EDTA at pH 8.0), supplemented with 1% SDS. To achieve further purification, the resuspended RNA was extracted twice using a phenol/chloroform/LET mixture. The purified RNA samples were then analyzed on 1.5% agarose gels containing 6.66% formaldehyde, and the RNA bands were successfully transferred onto Hybond-N membranes. After the transfer process, the nylon membrane was carefully removed from the system, rinsed with 2X SSC to remove any residual agarose gel fragments, and dried at room temperature, then the membrane was exposed to a UV crosslinker with a wavelength of 254 nm and an energy setting of 120 mJ/cm² for 30–45 s to immobilize the RNA onto the nylon membrane. For the detection of 18S RNA, one of the membranes was incubated with a biotin-labeled 18S probe (TTTCGCTCTGGTCCGTCTTG, the sequence was labeled using the Biotin 3’ End DNA Labeling Kit, Cat#R3106, Beyotime Institute of Biotechnology, Shanghai, China). Hybridization was performed using the Biotin Northern Blot Kit (Cat#R0219, Beyotime Institute of Biotechnology, Shanghai, China), and the results were visualized using the GelDoc XR gel imaging analysis system. For the ac4C assay, another membrane crosslinked with total RNA was blocked with non-fat milk and then treated with the anti-ac4C antibody (Cat#ab252215, Abcam, Shanghai, China; diluted 1:1000) for overnight. Following this, the membrane was incubated with a secondary antibody (Cat#SA00001-1, Proteintech, Wuhan, China) and the results were observed using the GelDoc XR gel imaging analysis system.

### Ac4C dot blot

Approximately 10 μg of RNA was denatured at 95 °C for a duration of 5 min and promptly chilled on ice. Subsequently, 2 μl of this RNA was applied to nitrocellulose membranes (Merck, Shanghai, China). These membranes were then cross-linked using ultraviolet light for 10 min and washed in 10 ml of wash buffer (composed of 1×TBS and 0.02% Tween-20) for 5 min. Next, the membranes were submerged in blocking buffer (consisting of 1×TBS, 0.02% Tween-20, and 5% non-fat milk) at room temperature for an hour. Following the removal of the blocking buffer, the membranes were incubated with an anti-ac4C antibody (Cat#ab252215, Abcam, Shanghai, China; diluted 1:1000) at 4 °C overnight. This antibody is capable of detecting in vitro transcribed RNAs containing ac4C modification. After incubation, the membranes were washed three times with 10 ml of wash buffer at room temperature. Subsequently, a secondary antibody was added and incubated at room temperature for an hour. The membranes were then washed four times with wash buffer. For detection, ECL reagent was poured onto the membranes and exposed in the dark. Additionally, as an internal control, the membranes, which had been photographed and washed were incubated in 0.02% methylene blue (Cat# M9140, Sigma-Aldrich, Shanghai, China) for 2 h and washed with RNase-Free water for another 2 h. Since methylene blue can directly bind to RNA or DNA, the level of ac4C could be calculated based on the ratio of the intensity of the HRP-labeled ac4C signal to the total RNA signal detected by methylene blue.

### Human lentivirus plasmid and adipose tissue-targeted mouse adeno-associated virus (AAV) plasmid construction

Lentivirus overexpression human NAT10 (LV-OE-hNAT10) plasmid, lentivirus negative human control (LV-OE-hNC) plasmid, lentivirus shRNA human NAT10 (LV-shRNA-hNAT10) and lentivirus shRNA human negative control (LV-shRNA-hNC) were designed and constructed by Genechem Co.,Ltd., Shanghai, China. In addition, overexpressed mouse NAT10 (OE-mNAT10, NM_153126.4) plasmid and overexpressed mouse negative control plasmid (OE-mNC) was constructed. The coding sequence (CDS) of mNAT10 was amplified in 3T3-L1 cells. The primer sequence was as follows: F:5-GTGGATCCGAGCTCGGTACCCGCCACCATGAATCGGAAGAAGGTGGATAACC-3’, R:5’-ATATTTTATTACCGGTTTAATTAACTACTTCTTTCGCTTCAGTTTCATATC-3’. KpnI and PacI were used as restriction enzymes, the amplification product was inserted into GV658 vector. The siRNA mNAT10 (siRNA-mNAT10), siRNA mKLF9 (siRNA-mKLF9) plasmid and siRNA mouse negative control (siRNA-mNC) plasmid was designed and synthetized by Sangon Biotech (Shanghai, China). The most effective sequence was verified by qPCR (sense sequence: 5’-GCUGGAUUUGUUCCUGUCUAUTT-3’, the antisense: 5’-AUAGACAGGAACAAAUCCAGCTT-3’). Adipose tissue-targeted adenovirus shRNA mNAT10 (AAV-shRNA-mNAT10) and AAV shRNA mouse negative control (AAV-shRNA-mNC) plasmid were designed and constructed by Genechem Co.,Ltd., Shanghai, China, the vector element sequence was FABP4p-EGFP-MIR155(MCS)-SV40 PolyA. In addition, the hNAT10 (G641E) mutation (MUT) and wild type (WT) plasmid were constructed by Genechem Co.,Ltd., Shanghai, China.

### Cell transfection

The transfection process was performed by using our previously established protocols [68, 69]. Briefly, 1 × 10^5^ hADSCs or 3T3-L1 cells were seeded in six-well plates and incubated for 24 h. Upon reaching a cell density of approximately 80%, the cells were transfected with lentiviral vectors, plasmids, or siRNA in a serum-free medium for a duration of 16 h. After transfection, the medium was replaced with a complete growth medium. Specifically, the hADSCs were transfected with LV-hNAT10, LV-hNC, LV-shRNA-hNAT10, LV-shRNA-hNC, and LV-hNAT10-mutation (mutated hNAT10), respectively. In parallel, 3T3-L1 cells were transfected with OE-mNAT10, OE-mNC, siRNA-mNAT10, siRNA-mNC, and OE-mNAT10 combined with siRNA-mKLF9, following the manufacturer’s instructions. Subsequently, the transfected cells were induced to differentiate into mature adipocytes.

### Ac4C-specific RNA immunoprecipitation and next generation sequencing (acRIP-seq) and acRIP-PCR

The acRIP-seq was performed and data was analyzed by Epibiotek Co., Ltd, Guangzhou, China. Briefly, the 3T3-L1 cells were transfected with siRNA-mNAT10 or siRNA-mNC for 48 h and further were induced and differentiated for 48 h. Total RNA was extracted from two groups, after rRNA was removed using QIAseq FastSelect-rRNA HMR Kits (Qiagen, Cat. No: 334385), about 200 μg RNA was fragmented using Epi^TM^ ac4C immunoprecipitation kit (Epibiotek Co., Ltd, Guangzhou, China, R1815) according to operating manual. The fragmented RNA was mixed with ac4C or IgG (Abcam, ab172730) antibody for 4 h at 4 °C; then, the protein A/G magnetic beads (Cat# 8880210002D/10004D, Invitrogen) were added and incubated for 2 h at 4 °C. The supernatant was discarded and the magnetic beads were washed for twice. The RNA was purified and precipitated, RNase inhibitor was added and incubated at 4 °C for overnight; then, the magnetic beads were washed for twice, the ac4C-enriched RNA was collected and RNA-seq library was constructed. Lastly, paired-end sequencing was performed in an Illumina NovaSeq 6000 sequencer (Illumina Inc., San Diego, CA, USA). The ac4C peaks were visualized by Integrative Genomics Viewe (IGV) software. In addition, hKLF9 and mKLF9 mRNA ac4C modification sites were also predicted by PACES software (http://www.rnanut.net/paces/). The mKLF9 acRIP-qPCR was amplified from cDNA which was reverse transfected by ac4C-enriched RNA. The premiers were designed with primer 5.0. The sequence of the primer 1 (included motif 1 and 2) was as follows: F: 5’-TGGCTCGCAGTTGGCTTT-3’, R: 5’-ACCTCAGCCCCTCATCTTTAC-3’; the sequence of the primer 2 (motif 3) was as follows: F: 5’-GTAAAGATGAGGGGCTGAGGT-3’, R: 5’-CCCTGGCCTCGGACGA-3’. The hKLF9 (NM_001206.4) acRIP-PCR primer was similar to mouse primer 2.

### RNA-seq

The 3T3-L1 cells were transfected with siRNA-mNAT10 or siRNA-mNC for 48 h and further were induced and differentiated for 48 h. Then, the RNA-seq was performed and data analysis was performed by Epibiotek Co., Ltd., Guangzhou, China. The differentially expressed genes (DEGs) was analyzed with DEseq2 software according |log2FC | > 1 and *P* value was <0.05. The GO analysis (https://geneontology.org/) and pathway analysis (https://www.kegg.jp/) were alos performed.

### NAT10 RIP-PCR

The NAT10 RIP according to acRIP method as mentioned above; the mRNAs combined to NAT10 were collected; the RNA was reverse transcribed and mKLF9 mRNA was amplified.

### Dual-luciferase reporter assay

According to the three ac4C motif sequences of mouse KLF9, pGL3-basic-mKLF9-mutation 1 (mKLF9-MUT1), pGL3-basic-mKLF9-mutation 2 (mKLF9-MUT2), pGL3-basic-mKLF9-mutation 3 (mKLF9-MUT3) and pGL3-basic-mKLF9-wild type (mKLF9-WT) plasmids were constructed, and dual-luciferase reporter assay was performed in 3T3-L1 cells. In addition, the ac4C motif sequences of hKLF9-wild type (hKLF9-WT) and pGL3-basic-hKLF9-mutation (hKLF9-MUT) plasmids were also constructed, dual-luciferase reporter assay was performed in HEK293T cells. Dual-luciferase reporter assay system (Cat# N1521, Promega, Madison, WA, USA) was used to detect luciferase activity. The double luciferase assay was provided by Jiman Biotechnology Co., LTD., Shanghai, China.

### mRNA stability assay

The 3T3-L1 cells were transfected with siRNA-mNAT10 and siRNA-mNC for 48 h, and the cells were then treated by 5 μg/mL Actinomycin D (S8964, Selleck, Houston, USA) for 0, 2, 4, 6 h, the KLF9 mRNA expression was amplified by RT-qPCR. The half-life(t_1/2_) was calculated according to the formula developed by Wang, et al. [30].

### CCK-8 assay

The cell viability was evaluated by CCK-8 (Cat# C0037, Beyotime Institute of Biotechnology, Shanghai, China). The 3T3-L1 cells were treated with 0, 5, 10, 20, and 40 μM Remodelin (*n* = 5); 10 μL CCK-8 was added into each well, and cells were incubated at 37 °C, 5%CO2 for 4 h. The absorbance was detected by microplate reader (Molecular Devices, Sunnyvale, USA) at 450 nm.

### Animal experiment

6-weeks-old male C57BL/6J mice were injected with adipose tissue targeted AAV-shRNA-mNAT10 (3 × 10^11^v.g, *n* = 5) or AAV-shRNA-mNC (3 × 10^11^v.g, *n* = 5) in left iWATs and physiological saline was injected into the right iWATs, respectively. Mice were fed with high-fat diet for 12 weeks. In addition, at 4th week, two groups of mice were injected with same dose AAV again in left iWATs. The NAT10 expression were detected with qPCR and western blotting. In addition, 6-week-old male C57BL/6J mice were treated with Remodelin (100 mg/kg/day, *n* = 5), a specific inhibitor of NAT10, or DMSO (*n* = 5) by gavage, respectively, the two groups also were fed by high-fat diet for 12 weeks.

### Hematoxylin and eosin (H&E) staining

The iWATs was fixed with 4% paraformaldehyde for 24 h, then, the iWATs were dehydrated and embedded with paraffin wax, subsequently, the wax blocks were cut about 5 μm-thick sections. After the sections were deparaffined and rehydrated, the H&E (Cat# G1120, Solarbio Science & Technology Co. LTD, Beijing, China) staining was performed according to the manufacturer’s protocol. The images were captured by microscope (Leica DM IL LED Fluo, Germany) and adipocyte size was analyzed with image processing software (Leica QWin V3, Cambridge, England).

### Glucose tolerance test (GTT) and insulin tolerance test (ITT)

After completing the 12-week AAV-mediated NAT10 adipose tissue-targeted silencing experiment, mice underwent a 12-h fasting period for GTT. In summary, they were intraperitoneally administered a glucose dose of 1.5 g/kg, and their blood glucose levels were meticulously monitored at various time points: 0, 30, 60, 90, and 120 min post-injection. Additionally, for the ITT mice were fasted for 12 h and then injected with 1 IU/kg of insulin sourced from Novo Nordisk (Copenhagen, Denmark).

### Statistics analysis

The data underwent analysis utilizing Graphpad Prism 10 software, the data from two groups were compared using a *t*-test, significance is defined as *p* < 0.05.

## Supplementary information

Supplementary table 1, 2 and figures

Supplementary table 3 and 4 -acRIP-seq+RNA-seq data

## Data Availability

The acRIP-seq and RNA-seq data are available in supplementary table 3 and 4.

## References

[1] Apovian CM, Guo XR, Hawley JA, Karmali S, Loos RJF, Waterlander WE. Approaches to addressing the rise in obesity levels. Nat Rev Endocrinol. 2023;19:76–81. 36450930 10.1038/s41574-022-00777-1

[2] Delaney KZ, Santosa S. Sex differences in regional adipose tissue depots pose different threats for the development of Type 2 diabetes in males and females. Obes Rev. 2022;23:e13393. 34985183 10.1111/obr.13393

[3] Ajoolabady A, Lebeaupin C, Wu NN, Kaufman RJ, Ren J. ER stress and inflammation crosstalk in obesity. Med Res Rev. 2023;43:5–30. 35975736 10.1002/med.21921

[4] Zhou Y, Li H, Xia N. The interplay between adipose tissue and vasculature: role of oxidative stress in obesity. Front Cardiovasc Med. 2021;8:650214. 33748199 10.3389/fcvm.2021.650214 PMC7969519

[5] Longo M, Zatterale F, Naderi J, Parrillo L, Formisano P, Raciti GA, et al. Adipose tissue dysfunction as determinant of obesity-associated metabolic complications. Int J Mol Sci. 2019;20:2358. 10.3390/ijms20092358 PMC6539070 31085992

[6] Boo SH, Kim YK. The emerging role of RNA modifications in the regulation of mRNA stability. Exp Mol Med. 2020;52:400–8. 32210357 10.1038/s12276-020-0407-z PMC7156397

[7] Roy B. Effects of mRNA modifications on translation: an overview. Methods Mol Biol. 2021;2298:327–56. 34085254 10.1007/978-1-0716-1374-0_20

[8] Liang W, Lin Z, Du C, Qiu D, Zhang Q. mRNA modification orchestrates cancer stem cell fate decisions. Mol Cancer. 2020;19:38. 32101138 10.1186/s12943-020-01166-w PMC7043046

[9] Abdollahi S, Hasanpour Ardekanizadeh N, Poorhosseini SM, Gholamalizadeh M, Roumi Z, Goodarzi MO, et al. Unraveling the complex interactions between the Fat Mass and Obesity-Associated (FTO) gene, lifestyle, and cancer. Adv Nutr. 2022;13:2406–19. 36104156 10.1093/advances/nmac101 PMC9776650

[10] Li J, Zhang H, Wang H. N(1)-methyladenosine modification in cancer biology: current status and future perspectives. Comput Struct Biotechnol J. 2022;20:6578–85. 36467585 10.1016/j.csbj.2022.11.045 PMC9712505

[11] Liu Y, Zhao Y, Wu R, Chen Y, Chen W, Liu Y, et al. mRNA m5C controls adipogenesis by promoting CDKN1A mRNA export and translation. RNA Biol. 2021;18:711–21. 34570675 10.1080/15476286.2021.1980694 PMC8782175

[12] Thalalla Gamage S, Howpay Manage SA, Chu TT, Meier JL. Cytidine Acetylation across the tree of life. Acc Chem Res. 2024;57:338–48. 38226431 10.1021/acs.accounts.3c00673 PMC11578069

[13] Ma CR, Liu N, Li H, Xu H, Zhou XL. Activity reconstitution of Kre33 and Tan1 reveals a molecular ruler mechanism in eukaryotic tRNA acetylation. Nucleic Acids Res. 2024;52:5226–40. 38613394 10.1093/nar/gkae262 PMC11109946

[14] Arango D, Sturgill D, Alhusaini N, Dillman AA, Sweet TJ, Hanson G, et al. Acetylation of cytidine in mRNA promotes translation efficiency. Cell. 2018;175:1872–86.e1824. 30449621 10.1016/j.cell.2018.10.030 PMC6295233

[15] Chen L, Wang WJ, Liu Q, Wu YK, Wu YW, Jiang Y, et al. NAT10-mediated N4-acetylcytidine modification is required for meiosis entry and progression in male germ cells. Nucleic Acids Res. 2022;50:10896–913. 35801907 10.1093/nar/gkac594 PMC9638909

[16] Sas-Chen A, Thomas JM, Matzov D, Taoka M, Nance KD, Nir R, et al. Dynamic RNA acetylation revealed by quantitative cross-evolutionary mapping. Nature. 2020;583:638–43. 32555463 10.1038/s41586-020-2418-2 PMC8130014

[17] Karthiya R, Wasil SM, Khandelia P. Emerging role of N4-acetylcytidine modification of RNA in gene regulation and cellular functions. Mol Biol Rep. 2020;47:9189–99. 33174082 10.1007/s11033-020-05963-w

[18] Wiener D, Schwartz S. The epitranscriptome beyond m(6)A. Nat Rev Genet. 2021;22:119–31. 33188361 10.1038/s41576-020-00295-8

[19] Arango D, Sturgill D, Yang R, Kanai T, Bauer P, Roy J, et al. Direct epitranscriptomic regulation of mammalian translation initiation through N4-acetylcytidine. Mol Cell. 2022;82:2797–814.e2711. 35679869 10.1016/j.molcel.2022.05.016 PMC9361928

[20] Xiang Y, Zhou C, Zeng Y, Guo Q, Huang J, Wu T, et al. NAT10-Mediated N4-Acetylcytidine of RNA contributes to post-transcriptional regulation of mouse oocyte maturation in vitro. Front Cell Dev Biol. 2021;9:704341. 34395433 10.3389/fcell.2021.704341 PMC8363255

[21] Chen X, Hao Y, Liu Y, Zhong S, You Y, Ao K, et al. NAT10/ac4C/FOXP1 promotes malignant progression and facilitates immunosuppression by reprogramming glycolytic metabolism in cervical cancer. Adv Sci. 2023;10:e2302705. 10.1002/advs.202302705 PMC10646273 37818745

[22] Yang Q, Lei X, He J, Peng Y, Zhang Y, Ling R, et al. N4-Acetylcytidine drives glycolysis addiction in gastric cancer via NAT10/SEPT9/HIF-1α positive feedback loop. Adv Sci. 2023;10:e2300898. 10.1002/advs.202300898 PMC10427357 37328448

[23] Yang W, Li HY, Wu YF, Mi RJ, Liu WZ, Shen X, et al. ac4C acetylation of RUNX2 catalyzed by NAT10 spurs osteogenesis of BMSCs and prevents ovariectomy-induced bone loss. Mol Ther Nucleic Acids. 2021;26:135–47. 34513300 10.1016/j.omtn.2021.06.022 PMC8413676

[24] Zhang M, Yang K, Wang QH, Xie L, Liu Q, Wei R, et al. The Cytidine N-Acetyltransferase NAT10 Participates in peripheral nerve injury-induced neuropathic pain by stabilizing SYT9 expression in primary sensory neurons. J Neurosci. 2023;43:3009–27. 36898834 10.1523/JNEUROSCI.2321-22.2023 PMC10146489

[25] Dalhat MH, Mohammed MRS, Alkhatabi HA, Rehan M, Ahmad A, Choudhry H, et al. NAT10: an RNA cytidine transferase regulates fatty acid metabolism in cancer cells. Clin Transl Med. 2022;12:e1045. 36149760 10.1002/ctm2.1045 PMC9505754

[26] Wu Y, Cao Y, Liu H, Yao M, Ma N, Zhang B. Remodelin, an inhibitor of NAT10, could suppress hypoxia-induced or constitutional expression of HIFs in cells. Mol Cell Biochem. 2020;472:19–31. 32529496 10.1007/s11010-020-03776-w

[27] Balmus G, Larrieu D, Barros AC, Collins C, Abrudan M, Demir M, et al. Targeting of NAT10 enhances healthspan in a mouse model of human accelerated aging syndrome. Nat Commun. 2018;9:1700. 29703891 10.1038/s41467-018-03770-3 PMC5923383

[28] Zhang Y, Deng Z, Sun S, Xie S, Jiang M, Chen B, et al. NAT10 acetylates BCL-XL mRNA to promote the proliferation of multiple myeloma cells through PI3K-AKT pathway. Front Oncol. 2022;12:967811. 35978804 10.3389/fonc.2022.967811 PMC9376478

[29] Zheng X, Wang Q, Zhou Y, Zhang D, Geng Y, Hu W, et al. N-acetyltransferase 10 promotes colon cancer progression by inhibiting ferroptosis through N4-acetylation and stabilization of ferroptosis suppressor protein 1 (FSP1) mRNA. Cancer Commun. 2022;42:1347–66. 10.1002/cac2.12363 PMC9759759 36209353

[30] Wang G, Zhang M, Zhang Y, Xie Y, Zou J, Zhong J, et al. NAT10-mediated mRNA N4-acetylcytidine modification promotes bladder cancer progression. Clin Transl Med. 2022;12:e738. 35522942 10.1002/ctm2.738 PMC9076013

[31] Zhang X, Chen J, Jiang S, He S, Bai Y, Zhu L, et al. N-Acetyltransferase 10 enhances doxorubicin resistance in human hepatocellular carcinoma cell lines by promoting the epithelial-to-mesenchymal transition. Oxid Med Cell Longev. 2019;2019:7561879. 31354912 10.1155/2019/7561879 PMC6636470

[32] Kimura H, Fujimori K. Activation of early phase of adipogenesis through Krüppel-like factor KLF9-mediated, enhanced expression of CCAAT/enhancer-binding protein β in 3T3-L1 cells. Gene. 2014;534:169–76. 24220850 10.1016/j.gene.2013.10.065

[33] Pei H, Yao Y, Yang Y, Liao K, Wu JR. Krüppel-like factor KLF9 regulates PPARγ transactivation at the middle stage of adipogenesis. Cell Death Differ. 2011;18:315–27. 20725087 10.1038/cdd.2010.100 PMC3131894

[34] Yan Q, Zhou J, Wang Z, Ding X, Ma X, Li W, et al. NAT10-dependent N(4)-acetylcytidine modification mediates PAN RNA stability, KSHV reactivation, and IFI16-related inflammasome activation. Nat Commun. 2023;14:6327. 37816771 10.1038/s41467-023-42135-3 PMC10564894

[35] Jin C, Wang T, Zhang D, Yang P, Zhang C, Peng W, et al. Acetyltransferase NAT10 regulates the Wnt/β-catenin signaling pathway to promote colorectal cancer progression via ac(4)C acetylation of KIF23 mRNA. J Exp Clin Cancer Res. 2022;41:345. 36522719 10.1186/s13046-022-02551-7 PMC9753290

[36] Wang Z, Wan X, Khan MA, Peng L, Sun X, Yi X, et al. NAT10 promotes liver lipogenesis in mouse through N4-acetylcytidine modification of Srebf1 and Scap mRNA. Lipids Health Dis. 2024;23:368. 39529018 10.1186/s12944-024-02360-1 PMC11552140

[37] Wang X, Wu R, Liu Y, Zhao Y, Bi Z, Yao Y, et al. m(6)A mRNA methylation controls autophagy and adipogenesis by targeting Atg5 and Atg7. Autophagy. 2020;16:1221–35. 31451060 10.1080/15548627.2019.1659617 PMC7469583

[38] Song T, Yang Y, Jiang S, Peng J. Novel insights into adipogenesis from the perspective of transcriptional and RNA N6-Methyladenosine-Mediated post-transcriptional regulation. Adv Sci. 2020;7:2001563. 10.1002/advs.202001563 PMC7610318 33173729

[39] Jiang X, Cheng Y, Zhu Y, Xu C, Li Q, Xing X, et al. Maternal NAT10 orchestrates oocyte meiotic cell-cycle progression and maturation in mice. Nat Commun. 2023;14:3729. 37349316 10.1038/s41467-023-39256-0 PMC10287700

[40] Uhlén M, Fagerberg L, Hallström BM, Lindskog C, Oksvold P, Mardinoglu A, et al. Proteomics. Tissue-based map of the human proteome. Science. 2015;347:1260419. 25613900 10.1126/science.1260419

[41] Xie L, Zhong X, Cao W, Liu J, Zu X, Chen L. Mechanisms of NAT10 as ac4C writer in diseases. Mol Ther Nucleic Acids. 2023;32:359–68. 37128278 10.1016/j.omtn.2023.03.023 PMC10148080

[42] Zhang Y, Lei Y, Dong Y, Chen S, Sun S, Zhou F, et al. Emerging roles of RNA ac4C modification and NAT10 in mammalian development and human diseases. Pharm Ther. 2024;253:108576. 10.1016/j.pharmthera.2023.108576 38065232

[43] Liu R, Wubulikasimu Z, Cai R, Meng F, Cui Q, Zhou Y, et al. NAT10-mediated N4-acetylcytidine mRNA modification regulates self-renewal in human embryonic stem cells. Nucleic Acids Res. 2023;51:8514–31. 37497776 10.1093/nar/gkad628 PMC10484679

[44] Ge J, Wang Z, Wu J. NAT10-mediated ac(4)C modification promotes ectoderm differentiation of human embryonic stem cells via acetylating NR2F1 mRNA. Cell Prolif. 2024;57:e13577. 38041497 10.1111/cpr.13577 PMC10984107

[45] Miao D, Shi J, Lv Q, Tan D, Zhao C, Xiong Z, et al. NAT10-mediated ac(4)C-modified ANKZF1 promotes tumor progression and lymphangiogenesis in clear-cell renal cell carcinoma by attenuating YWHAE-driven cytoplasmic retention of YAP1. Cancer Commun. 2024;44:361–83. 10.1002/cac2.12523 PMC10962679 38407929

[46] Liu Y, Wang X, Liu Y, Yang J, Mao W, Feng C, et al. N4-acetylcytidine-dependent GLMP mRNA stabilization by NAT10 promotes head and neck squamous cell carcinoma metastasis and remodels tumor microenvironment through MAPK/ERK signaling pathway. Cell Death Dis. 2023;14:712. 37914704 10.1038/s41419-023-06245-6 PMC10620198

[47] Pan Z, Bao Y, Hu M, Zhu Y, Tan C, Fan L, et al. Role of NAT10-mediated ac4C-modified HSP90AA1 RNA acetylation in ER stress-mediated metastasis and lenvatinib resistance in hepatocellular carcinoma. Cell Death Discov. 2023;9:56. 36765042 10.1038/s41420-023-01355-8 PMC9918514

[48] Hu Z, Lu Y, Cao J, Lin L, Chen X, Zhou Z, et al. N-acetyltransferase NAT10 controls cell fates via connecting mRNA cytidine acetylation to chromatin signaling. Sci Adv. 2024;10:eadh9871. 38215194 10.1126/sciadv.adh9871 PMC10786415

[49] Wang K, Zhou LY, Liu F, Lin L, Ju J, Tian PC, et al. PIWI-Interacting RNA HAAPIR Regulates Cardiomyocyte Death After Myocardial Infarction by Promoting NAT10-Mediated ac(4) C Acetylation of Tfec mRNA. Adv Sci. 2022;9:e2106058. 10.1002/advs.202106058 PMC8922123 35138696

[50] Zhang Y, Jing Y, Wang Y, Tang J, Zhu X, Jin WL, et al. NAT10 promotes gastric cancer metastasis via N4-acetylated COL5A1. Signal Transduct Target Ther. 2021;6:173. 33941767 10.1038/s41392-021-00489-4 PMC8093205

[51] Zhang H, Chen Z, Zhou J, Gu J, Wu H, Jiang Y, et al. NAT10 regulates neutrophil pyroptosis in sepsis via acetylating ULK1 RNA and activating STING pathway. Commun Biol. 2022;5:916. 36068299 10.1038/s42003-022-03868-x PMC9448771

[52] Shenshen W, Yin L, Han K, Jiang B, Meng Q, Aschner M, et al. NAT10 accelerates pulmonary fibrosis through N4-acetylated TGFB1-initiated epithelial-to-mesenchymal transition upon ambient fine particulate matter exposure. Environ Pollut. 2023;322:121149. 36731737 10.1016/j.envpol.2023.121149

[53] Qu R, Liu J, Feng L, Li L, Liu J, Sun F, et al. Down-regulation of KLF9 ameliorates LPS-caused acute lung injury and inflammation in mice via reducing GSDMD expression. Autoimmunity. 2022;55:587–96. 35993279 10.1080/08916934.2022.2114465

[54] Han N, Zhang L, Guo M, Yu L. Knockdown of Krüppel-Like Factor 9 Inhibits Aberrant Retinal Angiogenesis and Mitigates Proliferative Diabetic Retinopathy. Mol Biotechnol. 2023;65:612–23. 36109428 10.1007/s12033-022-00559-0

[55] Larrieu D, Britton S, Demir M, Rodriguez R, Jackson SP. Chemical inhibition of NAT10 corrects defects of laminopathic cells. Science. 2014;344:527–32. 24786082 10.1126/science.1252651 PMC4246063

[56] Dalhat MH, Mohammed MRS, Ahmad A, Khan MI, Choudhry H. Remodelin, a N-acetyltransferase 10 (NAT10) inhibitor, alters mitochondrial lipid metabolism in cancer cells. J Cell Biochem. 2021;122:1936–45. 34605570 10.1002/jcb.30155

[57] Shrimp JH, Jing Y, Gamage ST, Nelson KM, Han J, Bryson KM, et al. Remodelin is a cryptic assay interference chemotype that does not inhibit NAT10-Dependent cytidine acetylation. ACS Med Chem Lett. 2021;12:887–92. 34141066 10.1021/acsmedchemlett.0c00193 PMC8201477

[58] Dalhat MH, Altayb HN, Khan MI, Choudhry H. Structural insights of human N-acetyltransferase 10 and identification of its potential novel inhibitors. Sci Rep. 2021;11:6051. 33723305 10.1038/s41598-021-84908-0 PMC7960695

[59] Liu Y, Huang H, Zhang CB, Fan HN. N-acetyltransferase 10 promotes the progression of oral squamous cell carcinoma through N4-acetylcytidine RNA acetylation of MMP1 mRNA. Cancer Sci. 2023;114:4202–15. 37705232 10.1111/cas.15946 PMC10637085

[60] Deng M, Zhang L, Zheng W, Chen J, Du N, Li M, et al. Helicobacter pylori-induced NAT10 stabilizes MDM2 mRNA via RNA acetylation to facilitate gastric cancer progression. J Exp Clin Cancer Res. 2023;42:9. 36609449 10.1186/s13046-022-02586-w PMC9817303

[61] Zong G, Wang X, Guo X, Zhao Q, Wang C, Shen S, et al. NAT10-mediated AXL mRNA N4-acetylcytidine modification promotes pancreatic carcinoma progression. Exp Cell Res. 2023;428:113620. 37156457 10.1016/j.yexcr.2023.113620

[62] Shi J, Yang C, Zhang J, Zhao K, Li P, Kong C, et al. NAT10 is involved in cardiac remodeling through ac4C-mediated transcriptomic regulation. Circ Res. 2023;133:989–1002. 37955115 10.1161/CIRCRESAHA.122.322244

[63] Tsai K, Jaguva Vasudevan AA, Martinez Campos C, Emery A, Swanstrom R, Cullen BR. Acetylation of Cytidine residues boosts HIV-1 gene expression by increasing viral RNA stability. Cell Host Microbe. 2020;28:306–12.e306. 32533923 10.1016/j.chom.2020.05.011 PMC7429276

[64] Akimoto K, Miyata A, Kangawa K, Matsuo H, Koga Y, Matsuoka Y, et al. Plasma and right auricle concentrations of atrial natriuretic polypeptide in children with cardiac diseases. Eur J Pediatr. 1988;147:485–9. 2970389 10.1007/BF00441972

[65] Zuk PA, Zhu M, Mizuno H, Huang J, Futrell JW, Katz AJ, et al. Multilineage cells from human adipose tissue: implications for cell-based therapies. Tissue Eng. 2001;7:211–28. 11304456 10.1089/107632701300062859

[66] Fujita M, Matsumoto T, Hayashi S, Hashimoto S, Nakano N, Maeda T, et al. Paracrine effect of the stromal vascular fraction containing M2 macrophages on human chondrocytes through the Smad2/3 signaling pathway. J Cell Physiol. 2022;237:3627–39. 35766589 10.1002/jcp.30823

[67] Onoi Y, Matsumoto T, Anjiki K, Hayashi S, Nakano N, Kuroda Y, et al. Human uncultured adipose-derived stromal vascular fraction shows therapeutic potential against osteoarthritis in immunodeficient rats via direct effects of transplanted M2 macrophages. Stem Cell Res Ther. 2024;15:325. 39334434 10.1186/s13287-024-03946-3 PMC11438128

[68] Wan X, Zhu L, Zhao L, Peng L, Xiong J, Yang W, et al. hPER3 promotes adipogenesis via hHSP90AA1-mediated inhibition of Notch1 pathway. Cell Death Dis. 2021;12:301. 33741899 10.1038/s41419-021-03584-0 PMC7979882

[69] Wan X, Wang L, Khan MA, Peng L, Zhang K, Sun X, et al. Shift work promotes adipogenesis via cortisol-dependent downregulation of EGR3-HDAC6 pathway. Cell Death Discov. 2024;10:129. 38467615 10.1038/s41420-024-01904-9 PMC10928160

