# Supplementary material for: NAT10-mediated N4-acetylcytidine modification in KLF9 mRNA promotes adipogenesis
Source: Cell Death Differ. 2025 Mar 23;32(9):1613–29. doi: 10.1038/s41418-025-01483-x (PMC12432206; doi:10.1038/s41418-025-01483-x)
Supplement: Supplementary file 1 — Supplementary table 1, 2 and figures [file 41418_2025_1483_MOESM1_ESM.docx]

**Supplementary table 1. Clinicopathological features of normal body weight (control) and obese patients**

|  | Control-1 | Control-2 | Control-3 | Control-4 | Control-5 | Control-6 | Obese-1 | Obese-2 | Obese-3 | Obese-4 | Obese-5 | Obese-6 |
| --- | --- | --- | --- | --- | --- | --- | --- | --- | --- | --- | --- | --- |
| Gender | M | F | M | M | F | F | F | M | F | F | M | M |
| Age | 29 | 37 | 57 | 63 | 49 | 32 | 31 | 32 | 51 | 58 | 51 | 38 |
| BMI (kg/m^2^) | 20.8 | 23.3 | 21.7 | 22.1 | 20.2 | 21.9 | 33.6 | 34.1 | 36.5 | 32.7 | 35.9 | 36.2 |
| Bp(mmHg） | 126/70 | 131/66 | 143/93 | 147/90 | 123/76 | 135/73 | 137/65 | 153/86 | 163/97 | 142/64 | 152/87 | 158/82 |
| Cr(μmmol/L) | 81 | 76 | 59 | 76 | 61 | 78 | 59 | 72 | 62 | 73 | 78 | 60 |
| ALT(U/L) | 36 | 48 | 32 | 31 | 49 | 41 | 57 | 59 | 60 | 65 | 39 | 65 |
| AST(U/L) | 41 | 38 | 32 | 39 | 33 | 47 | 51 | 41 | 52 | 48 | 36 | 58 |

M = male, F = female, BMI = body mass index, Bp = blood pressure, Cr = creatinine, ALT = alanine aminotransferase, AST = aspartate transaminase

**Supplementary table 2. The sequences of primers for RT-qPCR**

| Gene name | Primer sequence (5’-3’) |
| --- | --- |
| hNAT10 | Forward:5’-CAGGCTTACTACGTGACCCG-3’ |
|  | Reverse:5’-TCTTCCGGTGACTGCGCC-3’ |
| mNAT10 | Forward:5’-GGGTAGCAACTCGGTGTCTT-3’ |
|  | Reverse5’-GAAAAGCTATCACTGCGCCC-3’ |
| hKLF9 | Forward:5’-GCCGCCTACATGGACTTCG-3’ |
|  | Reverse:5’-GGATGGGTCGGTACTTGTTCA-3’ |
| mKLF9 | Forward:5’-GCTGGTCACTATCGCCAAGA-3’ |
|  | Reverse:5’-TCCTCATCGGGACTCTCCAG-3’ |
| hPPARG | Forward:5’-GGGATCAGCTCCGTGGATCT-3’ |
|  | Reverse:5’-TGCACTTTGGTACTCTTGAAGTT-3’ |
| mPPARG | Forward:5’-GGAAGACCACTCGCATTCCTT-3’ |
|  | Reverse:5’-GTAATCAGCAACCATTGGGTCA-3’ |
| hCEBPA | Forward:5’-TGGACAAGAACAGCAACGAG-3’ |
|  | Reverse:5’-TTGTCACTGGTCAGCTCCAG-3’ |
| mCEBPA | Forward:5’-GCGGGAACGCAACAACATC-3’ |
|  | Reverse:5’-GTCACTGGTCAACTCCAGCAC-3’ |
| hFABP4 | Forward:5’-ACTGGGCCAGGAATTTGACG-3’ |
|  | Reverse:5’-CTCGTGGAAGTGACGCCTT-3’ |
| mFABP4 | Forward:5’-AAGGTGAAGAGCATCATAACCCT-3’ |
|  | Reverse:5’-TCACGCCTTTCATAACACATTCC-3’ |
| hUCP1 | Forward:5’-AGGATCGGCCTCTACGACAC-3’ |
|  | Reverse:5’-GCCCAATGAATACTGCCACTC-3’ |
| mUCP1 | Forward:5’-AGGCTTCCAGTACCATTAGGT-3’ |
|  | Reverse:5’-CTGAGTGAGGCAAAGCTGATTT-3’ |
| hGAPDH | Forward:5’-GGAGCGAGATCCCTCCAAAAT-3’ |
|  | Reverse:5’-GGCTGTTGTCATACTTCTCATGG-3’ |
| mGAPDH | Forward:5’-AGGTCGGTGTGAACGGATTTG-3’ |
|  | Reverse:5’-GGGGTCGTTGATGGCAACA-3’ |

**Supplementary Figure and legend**


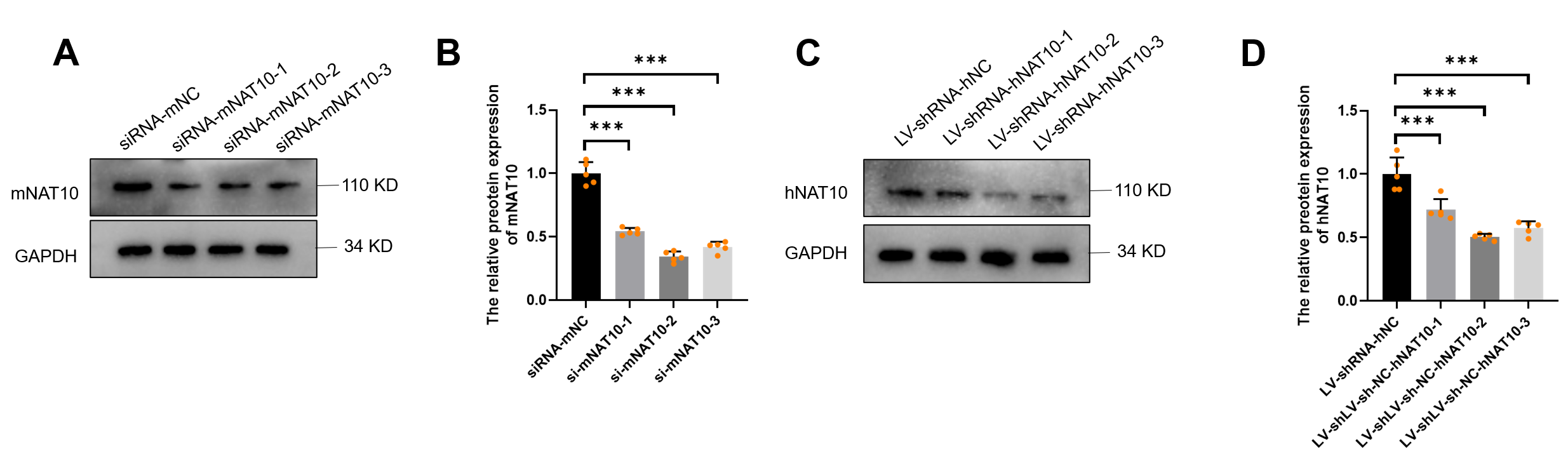


**Fig. S1 The transfection efficiency of NAT10 silencing**

**A, B** Three distinct pairs of silencing sequences were designed for mNAT10 and hNAT10 (**C, D**). Upon analysis using Western blot, it was found that siRNA-mNAT10-2 and LV-shRNA-hNAT10-2 demonstrated the utmost efficiency. Consequently, we chose these two silencing sequences for further experiments.


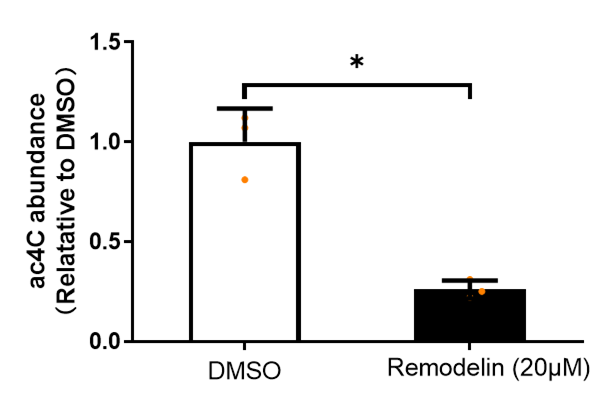


**Fig. S2** **The ac4C abundance was decreased in the Remodelin treated cells**

3T3-L1 cells were induced for 9 days, the culture medium contained 20 μM Remodelin or DMSO, the ac4C abundance quantitation and significance of difference were analyzed accordingly to Fig. 7N (n=3).


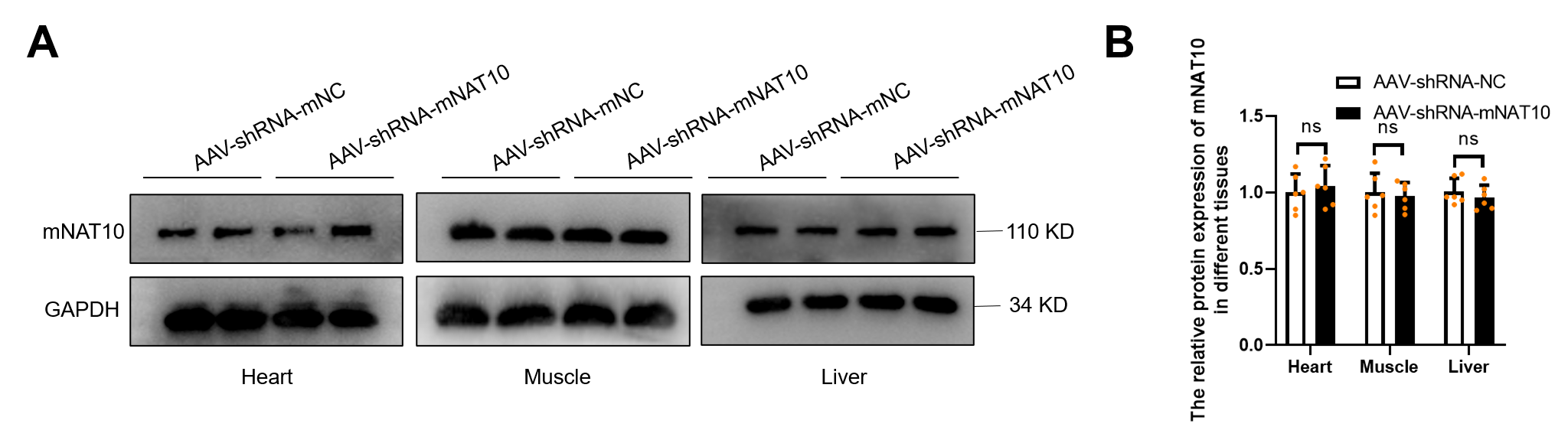


**Fig. S3 Investigation of tissue specificity with adipose tissue targeted AAV-shRNA-mNAT10 transfection**

**A, B** Adipose tissue targeted AAV-shRNA-mNAT10 exhibits remarkable tissue specificity, showing no reduction in the expression of mNAT10 in the heart, muscle, and liver (n=6).


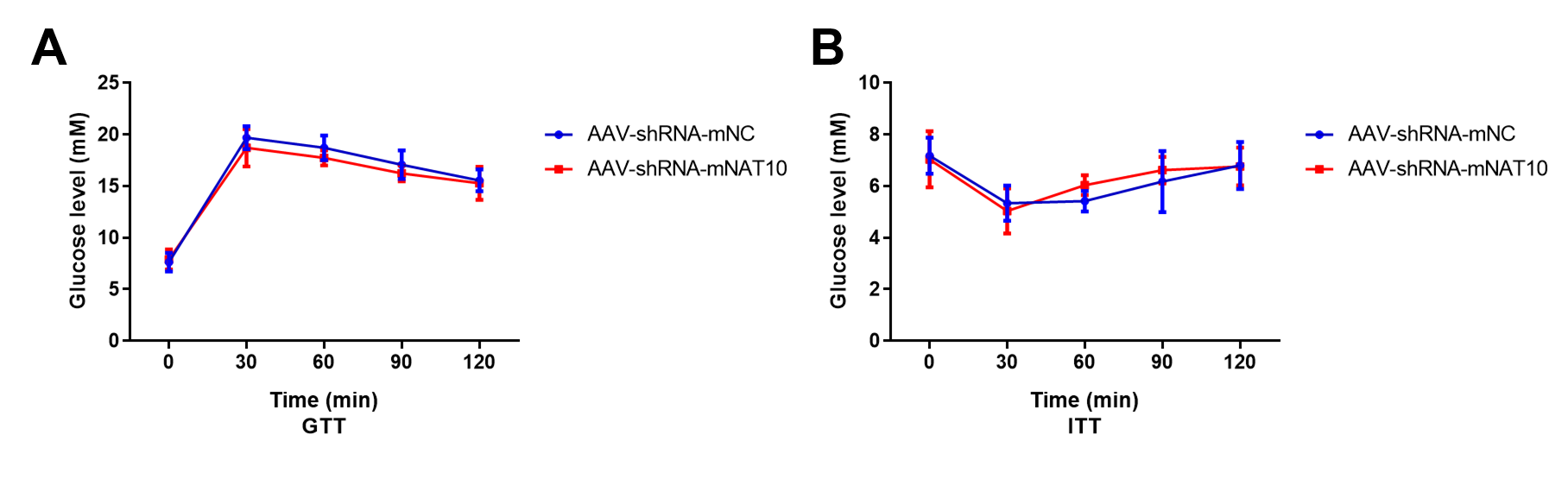


**Fig. S4 The efficiency of NAT10 silencing**

Mice were fed high-fat diet for 12 weeks and injected with adipose tissue targeted AAV-shRNA-mNAT10 (3×10^11^v.g, n=5) or AAV-shRNA-mNC (3×10^11^v.g, n=5) twice (0th and 4th week) in left iWATs. **A** GTT (n=5) and (**B**) ITT (n=5) were used to evaluated for glucose tolerance and insulin sensitivity, respectively.
